# First principles exploration of high hydrogen storage capacity of lithium-based perovskite hydrides

**DOI:** 10.1007/s00894-026-06861-x

**Published:** 2026-07-30

**Authors:** Fikadu Takele Geldasa, Francis Birhanu Dejene

**Affiliations:** Department of Chemical and Physical Sciences, Walter Sisulu University, Private Bag X1, Mthatha, 5117 South Africa

**Keywords:** Li-based hydride perovskites, Hydrogen storage, First-principles calculations, Mechanical and dynamical stability, Desorption temperature

## Abstract

**Context:**

The search for efficient solid-state hydrogen storage materials is essential for advancing clean energy technologies. In this work, Li-based perovskite hydrides (LiBH_3_, LiCuH_3_, LiMgH_3_, and LiSiH_3_) are studied using first-principles calculations. All compounds show negative formation energies, indicating thermodynamic stability. LiBH_3_, LiCuH_3_, and LiMgH_3_ are dynamically stable, while LiSiH_3_ is unstable due to imaginary phonon modes. Mechanical analysis confirms stability for all systems, with LiCuH_3_ and LiMgH_3_ showing higher rigidity. Electronic properties reveal metallic behavior for LiBH_3_, LiCuH_3_, and LiSiH_3_, while LiMgH_3_ is semiconducting with a 2.59 eV band gap. Hydrogen storage capacities are 14.56 wt% for LiBH_3_, 4.11 wt% for LiCuH_3_, 8.82 wt% for LiMgH_3_, and 7.95 wt% for LiSiH_3_. LiCuH_3_ also shows the lowest desorption temperature (382.7 K), indicating favorable kinetics.

**Methods:**

All calculations were performed within the framework of density functional theory (DFT) using the Quantum ESPRESSO package. The exchange–correlation energy was treated using the generalized gradient approximation in the Perdew–Burke–Ernzerhof (GGA-PBE) form, and electron–ion interactions were described using ultrasoft pseudopotentials. Valence configurations included Li (2s^1^), H (1s^1^), B (2s^2^2p^1^), Cu (3d^9.5^4s^1.5^), Mg (3s^2^), and Si (3s^2^3p^2^). Convergence tests determined a plane-wave cutoff energy of 55 Ry and a Monkhorst–Pack k-point mesh of 9 × 9 × 9, ensuring total energy convergence within 1–2 meV/atom. Structural optimizations were carried out using the Broyden–Fletcher–Goldfarb–Shanno (BFGS) algorithm with convergence thresholds of 1 × 10⁻^6^ eV/atom for energy and 1 × 10⁻^3^ eV/Å for forces. Phonon dispersion, mechanical properties, and optical responses were evaluated using the *thermo_pw* package integrated with Quantum ESPRESSO.

## Introduction

Hydrogen energy has emerged as one of the most promising pathways for transitioning toward clean, sustainable, and renewable energy systems. Its exceptionally high gravimetric energy density, lightweight nature, long-term storage stability, and capacity for large-scale utilization make hydrogen an attractive energy vector for future decarbonized technologies [1–3]. Moreover, hydrogen combustion produces only water, with no CO_2_ or other greenhouse gas emissions, offering a clear advantage over fossil fuels in achieving climate change mitigation and long-term carbon neutrality goals [4, 5]. The realization of a hydrogen-based energy infrastructure, however, critically depends on the development of efficient, safe, and compact hydrogen storage technologies. The current hydrogen storage strategies are compressed gas, cryogenic liquid, and solid-state systems, each provide unique benefits but also face limitations. Physical storage methods such as compression and liquefaction are technologically mature, yet suffer from high energy consumption, costly infrastructure, low volumetric efficiency, and inherent safety challenges [6, 7]. In contrast, solid-state hydrogen storage, where hydrogen is retained within a host material via physisorption or chemisorption, offers improved safety, reversibility, and compactness under moderate operating conditions [8].

Physisorption-based materials such as activated carbon, carbon nanotubes, fullerenes, zeolites, COFs, MOFs, and polymers of intrinsic microporosity (PIMs) provide fast adsorption kinetics, though their weak intermolecular interactions lead to low storage capacity at ambient conditions [9, 10]. Chemisorption-based systems, particularly metal hydrides, overcome this issue by forming stronger metal–hydrogen bonds, enabling significantly higher volumetric hydrogen densities than physical storage methods [11, 12]. Metal hydrides are widely recognized for their reversibility, intrinsic safety, and moderate operating conditions, storing hydrogen within well-defined lattice sites that minimize leakage and degradation [13]. Nevertheless, conventional binary hydrides such as FeTi, Mg_2_Ni, and LaNi_5_ exhibit relatively low gravimetric capacities (1.89–3.59 wt%) [10, 14, 15], and complex hydrides such as NaAlH_4_ and Mg(NH_2_)_2_–LiH, despite their higher capacities, often suffer from poor reversibility and multi-step dehydrogenation pathways [16, 17]. These challenges have intensified efforts to develop new classes of hydrides capable of providing high hydrogen storage densities while maintaining practical thermodynamic and kinetic properties.

In this context, hydride perovskites (ABH_3_-type compounds) have emerged as an exciting class of materials with significant potential for next-generation hydrogen storage systems. These materials exhibit unique crystal chemistry, combining high gravimetric and volumetric hydrogen densities with mechanical robustness and thermodynamic stability, while also supporting reversible hydrogen uptake and release under relatively mild conditions [18, 19]. Their perovskite crystal framework where atom A occupies the cube corners, atom B sits at the body center, and hydrogen atoms reside at the face-centered sites provides structural flexibility and multiple avenues for tailoring hydrogen storage performance [20, 21]. The wide tunability of A- and B-site elements enables systematic control of bonding strength, stability, diffusion pathways, and dehydrogenation energetics, offering distinct advantages over conventional metal hydrides with fixed stoichiometry.

Interest in perovskite hydrides has grown rapidly, driven by increasing experimental efforts and significant advances in computational materials science. Early pioneering work by Ronnebro et al. demonstrated the successful synthesis of NaMgH_3_ and identified its orthorhombic structure using single-crystal X-ray diffraction, thereby establishing a foundation for further exploration of perovskite hydrides [22]. Subsequent synthesis methods including high-pressure synthesis [23], mechanical milling [24], and solid-state reactions [25] enabled the preparation of various Mg-based perovskite hydrides. Although these materials exhibit high hydrogen capacities, their strong thermodynamic stability leads to elevated desorption temperatures, limiting their practical use for onboard hydrogen storage applications [26].

The rapid expansion of research on hydride perovskites is largely attributed to density functional theory (DFT), which has become indispensable for predicting stability, decoding hydrogen absorption/desorption mechanisms, and guiding experimental design [27]. A growing number of perovskite hydrides have been proposed through first-principles studies, many of which display highly attractive hydrogen storage characteristics. Notable examples include NaMgH_3_, widely investigated for its crystal chemistry, hydrogen mobility, and thermodynamic behavior [28]; LiNiH_3_, initially predicted through first-principles calculations and later successfully synthesized [29]; and broader compositional families such as NaXH_3_ (X = Mn, Fe, Co, Cr) [30] and MgXH_3_ (X = Cr, Fe, Mn) [31]. These studies collectively highlight the potential of perovskite hydrides for meeting technologically relevant hydrogen storage targets.

Motivated by the promising performance of Li-based hydride perovskites, recent DFT investigations have explored compounds such as LiCaH_3_ [32], LiSH_3_ [33], LiAlH_3_ [34], LiPH_3_ [6], and LiTiH_3_ [35], which demonstrate gravimetric storage capacities ranging from 5.5 wt% to 7.57 wt%. Reported hydrogen storage capacities of Li-based perovskites such as LiXH_3_ (X = Mn, Fe, Co: 4.4–4.6 wt%) and LiAH_3_ (A = Sc, Ti, V: 4.8–5.7 wt%), along with the 6 wt% capacity of NaMgH_3_, further illustrate their potential to meet or approach the U.S. DOE 2025 hydrogen storage targets [36, 37]. However, despite these individual studies, a systematic first-principles comparison of Li-based hydride perovskites incorporating elements from different groups of the periodic table remains lacking.

In the present study, we address this gap by performing a comprehensive first-principles investigation of the structural, dynamical, mechanical, thermodynamic, electronic, optical, and hydrogen storage properties of four Li-based hydride perovskites LiBH_3_, LiCuH_3_, LiMgH_3_, and LiSiH_3_. These compounds were deliberately selected to represent chemically diverse elements: B (non-metal), Cu (transition metal), Mg (alkaline-earth metal), and Si (semiconducting group-14 element). This strategic chemical diversity enables a meaningful comparative assessment of how A-site and B-site chemistry influences hydrogen storage capability. Each compound was modeled, optimized, and evaluated through detailed DFT analyses to elucidate stability, bonding, mechanical resilience, thermodynamic behavior, electronic structure, optical response, and hydrogen storage performance. The results of this study not only provide a unified framework for understanding Li-based hydride perovskites but also offer valuable guidance for the theoretical discovery and experimental realization of next-generation perovskite hydrides for hydrogen energy storage applications.

## Calculation methods

**Fig. 1:**
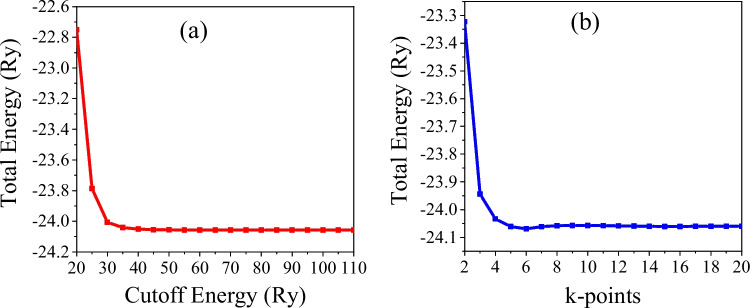
The convergence test of total energy versus (**a**) cutoff energy and (**b**) k-points for the representative hydride perovskite LiBH_3_

All calculations in this study were carried out within the framework of first-principles density functional theory (DFT) using the Quantum ESPRESSO package [38]. The initial crystal structure of LiBH_3_ was constructed in the cubic phase with a lattice parameter of *a* = 3.88947 Å and angles α = β = γ = 90°. The structures of LiCuH_3_, LiMgH_3_, and LiSiH_3_ were subsequently generated by substituting the B-site atom in LiBH_3_ with Cu, Mg, and Si, respectively. The exchange–correlation interactions were described using the generalized gradient approximation (GGA) within the Perdew–Burke–Ernzerhof functional (PBE) functional [39]. The electron–ion interactions were treated using ultrasoft pseudopotentials. Specifically, Li (li_pbe_v1.4.uspp.F.UPF), H (H.pbe-rrkjus_psl.1.0.0.UPF), and B (b_pbe_v1.4.uspp.F.UPF) pseudopotentials were obtained from the Standard Solid-State Pseudopotentials (SSSP) library, while Cu (Cu.pbe-dn-rrkjus_psl.0.2.UPF) and Mg (Mg.pbe-spnl-rrkjus_psl.1.0.0.UPF) were taken from the PSlibrary. The valence electron configurations considered were Li (2s^1^), H (1s^1^), B (2s^2^2p^1^), Cu (3d^9.5^4s^1.5^), Mg (3s^2^), and Si (3s^2^3p^2^). To ensure the accuracy and reliability of the calculations, convergence tests were performed with respect to both the plane-wave cutoff energy and k-point sampling using LiBH_3_ as a representative system. The kinetic energy cutoff was varied from 20 to 110 Ry in increments of 5 Ry. As shown in Fig. 1a, a cutoff energy of 55 Ry was sufficient to converge the total energy within 2 meV/atom. Brillouin zone integrations were carried out using Monkhorst–Pack k-point grids, with densities ranging from 2 × 2 × 2 to 20 × 20 × 20. As illustrated in Fig. 1b, a 9 × 9 × 9 k-point grid ensured convergence of the total energy within 1 meV/atom, providing an optimal compromise between computational cost and accuracy. These optimized parameters were employed consistently in all subsequent calculations. Structural optimization was performed using the Broyden–Fletcher–Goldfarb–Shanno algorithm (BFGS) minimization scheme. The convergence thresholds were set to 1 × 10^–6^ eV/atom for total energy and 1 × 10^–3^ eV/Å for atomic forces. Following full relaxation, the optimized lattice parameters and atomic positions were used for all further calculations. To investigate the mechanical, thermodynamic, and optical properties of the studied systems, the *thermo_pw* package integrated within Quantum ESPRESSO was employed.

## Results and discussion

### Structural characteristics

**Fig. 2:**
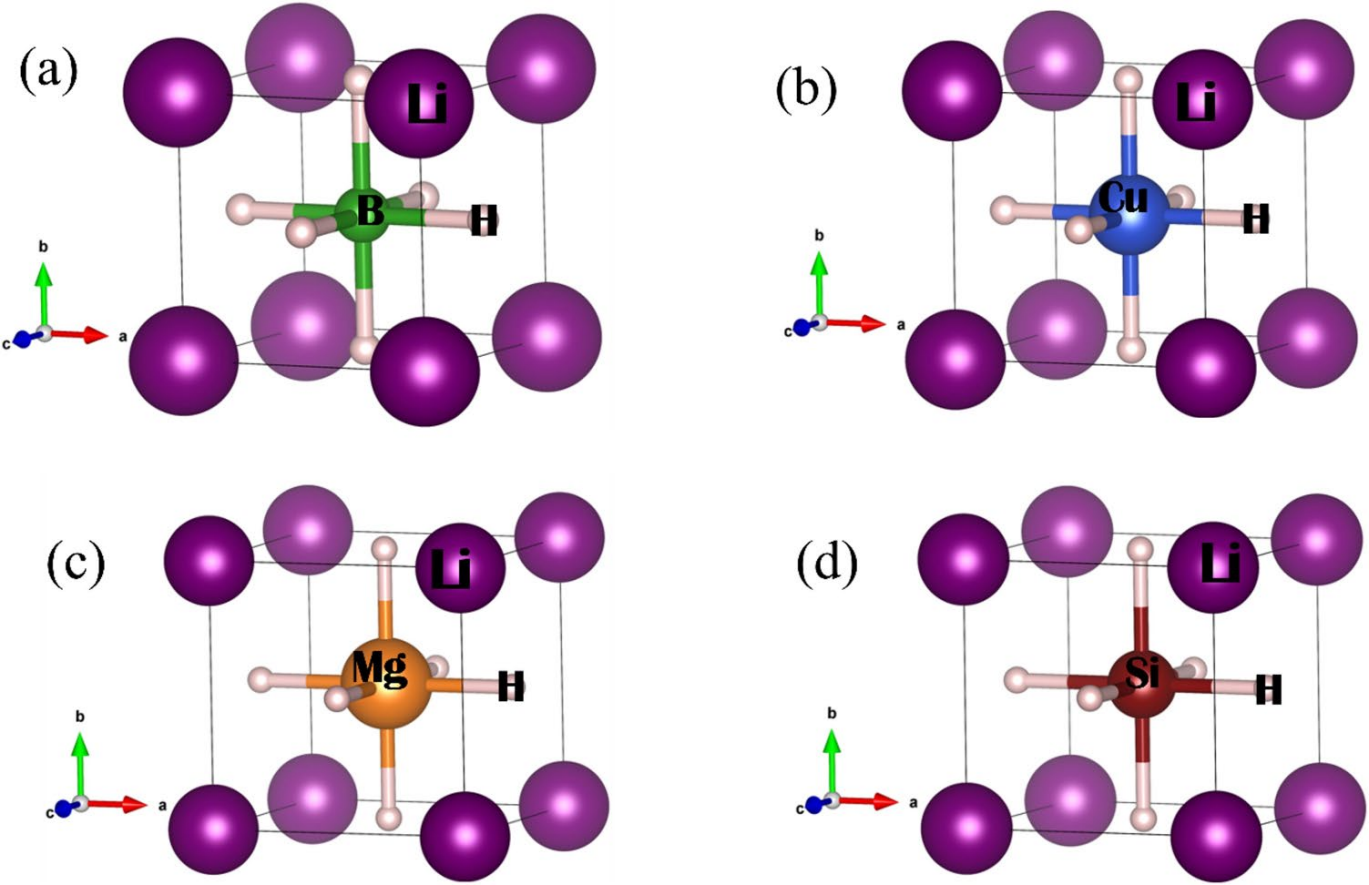
The optimized crystal structures of (**a**) LiBH_3_, (**b**) LiCuH_3_, (**c**) LiMgH_3_ and (**d**) LiSiH_3_

The Li-based hydride perovskites LiXH_3_ (X = B, Cu, Mg, Si) investigated in this study adopt a cubic crystal structure with a *Pm-3 m* space group following full geometry optimization. The optimized structures are depicted in Fig. 2(a–d). The atomic positions are defined by fractional coordinates: Li atoms occupy the corners at (0, 0, 0), B/Cu/Mg/Si atoms reside at the body center (0.5, 0.5, 0.5), and hydrogen atoms are located at the face-centered octahedral sites (0.5, 0.5, 0), (0.5, 0, 0.5), and (0, 0.5, 0.5).

The optimized lattice constants for LiBH_3_, LiCuH_3_, LiMgH_3_, and LiSiH_3_ were calculated to be 3.087 Å, 3.369 Å, 3.756 Å, and 3.691 Å, respectively as presented in Table 1. These variations in lattice constants can be attributed to the differences in ionic radii of B, Cu, Mg, and Si atoms, which directly affect bond lengths and the overall unit cell volumes [40]. Specifically, the B–H bond length in LiBH_3_ is 1.544 Å, whereas the Cu–H, Mg–H, and Si–H bond lengths in LiCuH_3_, LiMgH_3_, and LiSiH_3_ are 1.685 Å, 1.878 Å, and 1.846 Å, respectively. The longer bond lengths observed in LiMgH_3_ reflect the comparatively larger ionic radius of Mg, leading to an expanded unit cell volume relative to the other hydrides as presented in Table 1. Currently, no experimental data exist for LiBH_3_, LiCuH_3_, LiMgH_3_, or LiSiH_3_. Therefore, the present results were compared with previous DFT studies on similar Li-based perovskite hydrides (Table 1). While lattice constants for LiTiH_3_ and LiPH_3_ are considerably larger than those reported here, the calculated values for other Li-based hydrides show excellent agreement with prior theoretical reports. Discrepancies in lattice parameters are primarily attributed to differences in atomic ionic radii.

**Table 1:** Comparison of the obtained parameters in the present work with reported Li based perovskite hydrides

Hydrides	a (Å)	V_0_ (Å)^3^	B_0_ (GPa)	B_0_’	${E}_{f}$(eV/atom)	${E}_{coh}$(eV/atom)	Ref
LiBH_3_	3.087	29.45	86.8	3.35	− 0.76	0.70	This work
LiCuH_3_	3.369	38.27	73.1	3.92	− 0.62	0.62	This work
LiMgH_3_	3.756	52.99	38.9	3.44	− 0.69	0.69	This work
LiSiH_3_	3.691	50.30	47.3	2.56	− 0.63	0.63	This work
LiTiH_3_	6.95	−	64.52	3.62	–	–	[42]
LiRhH_3_	3.387	38.855	109.9	2.02	− 0.56	−	[43]
LiScH_3_	3.864	57.699	−	−	− 1.53	−	[35]
LiCaH_3_	4.291	−	−	−	– 0.089	−	[44]
LiSrH_3_	4.647	100.411	−	−	− 2.902	2.902	[45]
LiPH_3_	7.90	53.45	56.2	−	− 5.16	−	[6]
LiInH_3_	3.87	57.96	−	−	−	−	[46]
LiSH_3_	4.05	−	−	−	−	−	[33]
LiInH_3_	4.09	68.53	35.1	4.04	− 0.859	−	[34]
LiGaH_3_	3.74	52.61	44.1	3.54	− 1.064	−	[34]
LiAlH_3_	3.68	49.94	37.3	3.29	− 0.927	−	[34]
LiZnH_3_	3.37	38.38	53.92	4.0	− 0.82	−	[47]
LiVH_3_	3.451	277.21	80.15	4.62	− 248.86	−	[48]

**Fig. 3:**
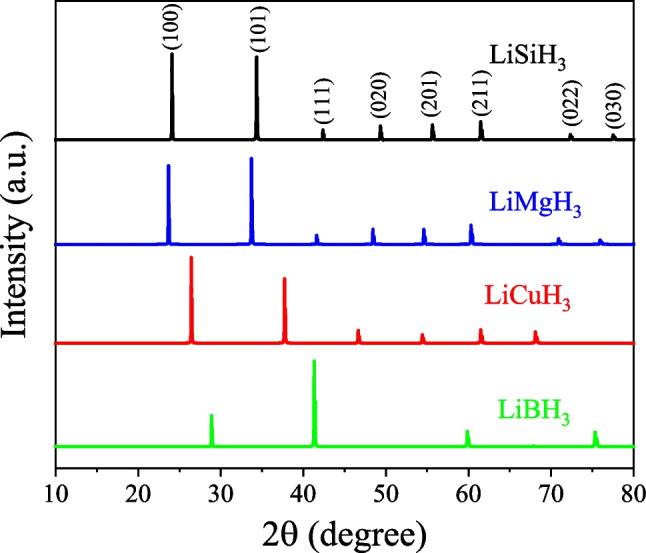
The simulated XRD patterns for LiBH_3_, LiCuH_3_, LiMgH_3_, and LiSiH_3_ hydrides

The simulated X-ray diffraction (XRD) patterns, obtained using VESTA software (Fig. 3), reveal that the dominant (100) and (101) diffraction peaks shift toward lower angles for hydrides with larger lattice constants and toward higher angles for those with smaller lattice constants [41]. Peak shifts toward lower angles indicate lattice expansion, whereas shifts toward higher angles indicate contraction.

A smaller lattice constant corresponds to a reduced unit cell volume, implying that the material occupies less physical space while storing an equivalent number of hydrogen atoms. This enhances volumetric hydrogen density, which is a critical parameter for practical hydrogen storage applications, including fuel cells and vehicular systems [27]. Moreover, lattice dimensions influence hydrogen diffusion pathways, bonding strength, and thermodynamic stability, all of which govern the kinetics and reversibility of hydrogen absorption and desorption.

Using the Birch–Murnaghan equation of state (EOS), equilibrium properties including total energy at the ground state (${E}_{0}$), equilibrium volume (${V}_{0}$), bulk modulus (${B}_{0}$), and pressure derivative bulk modulus (${B}{\prime}$) were obtained [45]:

1 $$E\left(V\right)={E}_{0}\left(V\right)+\frac{{B}_{0}V}{{B}{\prime}\left({B}{\prime}-1\right)}\left\{{B}_{0}\left(1-\frac{{V}_{0}}{V}\right)+{\left(\frac{{V}_{0}}{V}\right)}^{{B}{\prime}}-1\right\}$$

**Fig. 4:**
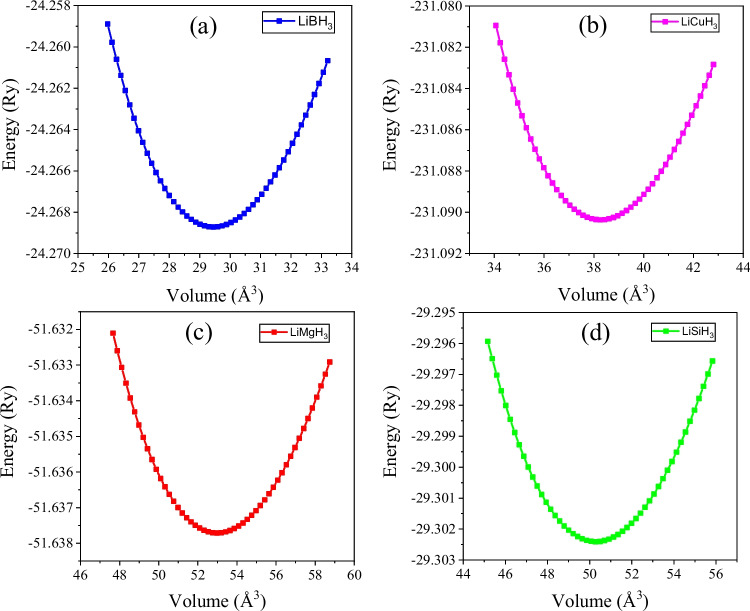
Total energy as function of volume for (**a**) LiBH_3_, (**b**) LiCuH_3_, (**c**) LiMgH_3_, and (**d**) LiSiH_3_ perovskite hydrides

The total energy as a function of volume for the four hydrides is illustrated in Fig. 4(a–d), with the calculated ${E}_{0}$, ${V}_{0}$, ${B}_{0}$, and ${B}{\prime}$ listed in Table 1. As expected, unit cell volume increases with increasing lattice constant, consistent with the atomic size of B, Cu, Mg, and Si. Among all hydrides, LiCuH_3_ exhibits the lowest ground-state total energy, indicating higher thermodynamic stability relative to the others. Furthermore, hydrides with larger volumes display smaller bulk moduli, confirming an inverse relationship between volume and stiffness.

### Formation energy

Evaluating the formation energy (${E}_{f}$) of hydride perovskites is essential for assessing their structural stability and suitability as hydrogen storage materials [49, 50]. Formation energy quantifies the minimum energy required to synthesize a compound from its constituent elements in their most stable states [51]. For the Li-based hydrides, ${E}_{f}$ is calculated as [52]:

2 $${E}_{f}={E}_{tot}\left(LiB{H}_{3}\right)-\left({E}_{Li}+{E}_{B}+\frac{3}{2}{E}_{{H}_{2}}\right)$$

3 $${E}_{f}={E}_{tot}\left(LiCu{H}_{3}\right)-\left({E}_{Li}+{E}_{Cu}+\frac{3}{2}{E}_{{H}_{2}}\right)$$

4 $${E}_{f}={E}_{tot}\left(LiMg{H}_{3}\right)-\left({E}_{Li}+{E}_{Mg}+\frac{3}{2}{E}_{{H}_{2}}\right)$$

5 $${E}_{f}={E}_{tot}\left(LiSi{H}_{3}\right)-\left({E}_{Li}+{E}_{Si}+\frac{3}{2}{E}_{{H}_{2}}\right)$$

where, ${E}_{tot}\left(LiB{H}_{3}\right)$, ${E}_{tot}\left(LiCu{H}_{3}\right)$, ${E}_{tot}\left(LiMg{H}_{3}\right)$ and ${E}_{tot}\left(LiSi{H}_{3}\right)$ is total energy of perovskite hydrides, ${E}_{Li}$ total energy of Li atoms, ${E}_{B}$, ${E}_{Cu}$, ${E}_{Mg}$ and ${E}_{Si}$ total energy of B, Cu, Mg, and Si atoms and ${E}_{{H}_{2}}$ is total energy of hydrogen molecules.

The calculated formation energies (Table 1) are all negative, confirming that LiBH_3_, LiCuH_3_, LiMgH_3_, and LiSiH_3_ are thermodynamically stable and potentially synthesizable [30]. LiBH_3_ exhibits the most negative formation energy, suggesting it is slightly more stable than the other hydrides.

For hydrogen storage applications, an optimal formation energy is crucial: if the hydrogen–host interaction is too weak (low formation energy), hydrogen desorbs too easily; if too strong (high formation energy), excessive energy is required for release [53]. The intermediate formation energies of these Li-based hydrides indicate that they can facilitate reversible hydrogen absorption and desorption under practical conditions.

The average atomic cohesive energy (${E}_{coh}$) provides a quantitative measure of atomic bonding strength within the crystal lattice. It is defined as the energy required to separate a solid into its constituent atoms, normalized by the total number of atoms [27]:

6 $${E}_{coh}=\left({E}_{A}+{E}_{B}+3/2{E}_{{H}_{2}}\right)-{E}_{AB{H}_{3}}$$

The calculated cohesive energies (Table 1) show that LiBH_3_ possesses the highest ${E}_{coh}$, reflecting the strongest overall bonding. LiCuH_3_ and LiSiH_3_ exhibit slightly lower but still substantial cohesive energies. This trend mirrors the formation energies and supports the conclusion that LiBH_3_ has the strongest B–hydrogen interactions among the studied hydrides.

### Molecular dynamics simulations

**Fig. 5:**
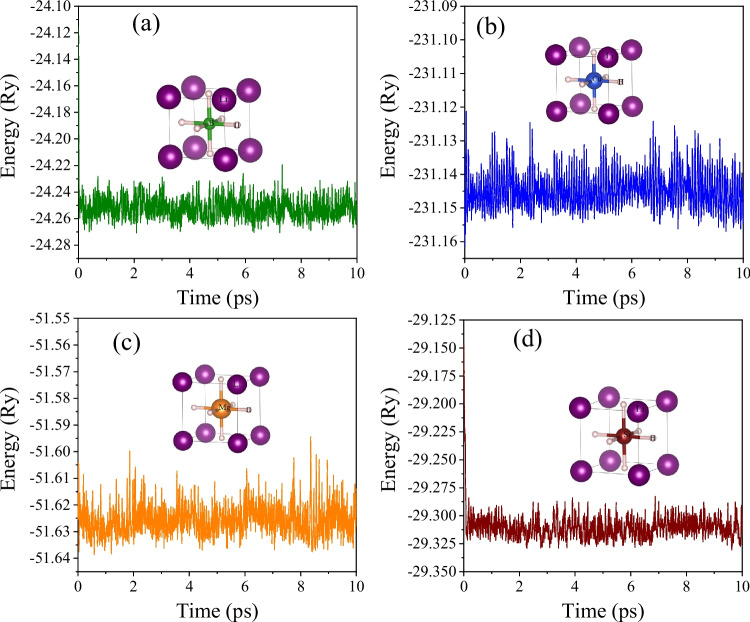
The AIMD simulations for LiBH_3_, LiCuH_3_, LiMgH_3_, and LiSiH_3_ perovskite hydrides at 500 K

The thermal stability of hydride perovskites is a key factor determining their feasibility for hydrogen storage applications, particularly under elevated operating temperatures. To further evaluate the thermal and structural robustness of the investigated compounds, ab initio molecular dynamics (AIMD) simulations were performed. The AIMD simulation results for LiBH_3_, LiCuH_3_, LiMgH_3_, and LiSiH_3_ perovskite hydrides are presented in Fig. 5(a–d). All simulations were conducted at 500 K for a total simulation time of 10 ps, corresponding to 10,000 iterations within 1 fs step. This temperature was chosen to assess stability well above ambient conditions and to emulate realistic hydrogen storage environments. The energy fluctuations remain relatively stable throughout the simulations, indicating that no significant structural distortions or bond breakages occurred. Compered to LiBH_3_ and LiSiH_3_, the energy fluctuations in LiCuH_3_ and LiMgH_3_ shows slightly greater relatively which may be attributed to the Cu and Mg metallic characteristics. Nevertheless, the absence of any sharp or linear energy rise signifies that no phase transition or structural degradation occurs throughout the simulation period. These results confirm that the studied hydrides possess substantial thermal and dynamic stability, underscoring their potential suitability for hydrogen storage [54].

### Phonon dispersion

**Fig. 6:**
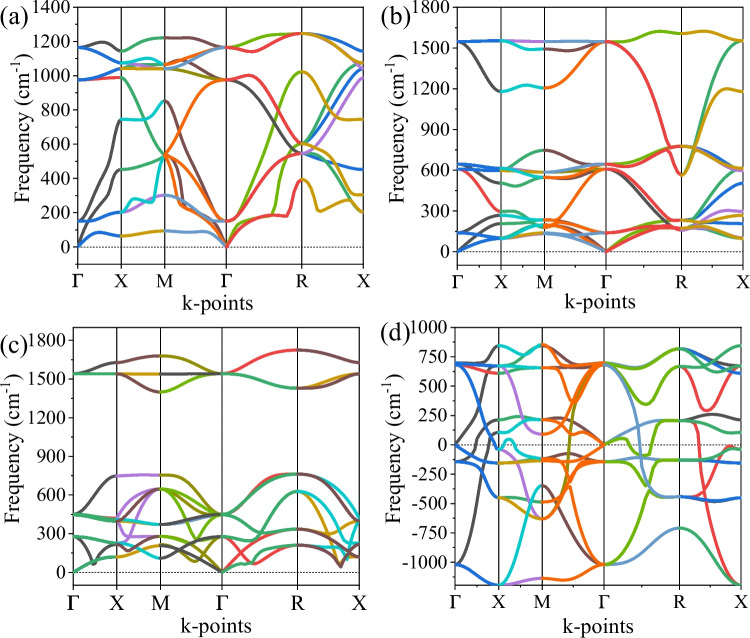
Phonon dispersion plots for (**a**) LiBH_3_, (**b**) LiCuH_3_, (**c**) LiMgH_3_, and (**d**) LiSiH_3_ perovskite hydrides

The dynamical stability of the Li-based hydride perovskites was further investigated through phonon dispersion calculations, which provide direct insight into lattice vibrational behaviour and structural stability. The phonon dispersion curves for LiBH_3_, LiCuH_3_, LiMgH_3_, and LiSiH_3_ are presented in Fig. 6(a–d). Phonon calculations are a crucial tool for assessing whether a crystal structure is dynamically stable, as they reveal how the lattice responds to small atomic displacements across the Brillouin zone [55]. Each phonon dispersion spectrum consists of acoustic and optical branches. The acoustic modes appear in the low-frequency region and correspond to collective, in-phase atomic vibrations analogous to sound-wave propagation through the lattice [56, 57]. In contrast, the optical modes occur at higher frequencies and originate from out-of-phase oscillations of neighbouring atoms. Since the primitive unit cell of LiXH_3_ (X = B, Cu, Mg, Si) contains five atoms, a total of 15 phonon modes are expected: three acoustic and twelve optical branches.

A fundamental criterion for dynamical stability is that all phonon frequencies must be positive (real) throughout the entire Brillouin zone [58]. Conversely, the presence of imaginary (negative) phonon frequencies signals dynamical instability, suggesting that the structure may undergo distortion, lattice reconstruction, or phase transformation [59]. Among the investigated compounds, LiBH_3_, LiCuH_3_, and LiMgH_3_ exhibit phonon dispersion curves entirely free of imaginary frequencies, confirming its dynamical and kinetic stability. This result indicates that LiBH_3_, LiCuH_3_, and LiMgH_3_ are structurally robust and potentially synthesizable, making it a particularly promising candidate for hydrogen storage applications.

In contrast, the phonon spectra of LiSiH_3_ exhibits imaginary phonon modes in certain regions of the Brillouin zone. The presence of these modes suggests that, although these structures may be metastable at finite temperatures as supported by AIMD simulations it is dynamically unstable and may require external stabilization mechanisms such as temperature effects, pressure, strain engineering, or chemical substitution to achieve full dynamical stability.

### Mechanical properties

Mechanical stability is a prerequisite for the practical deployment of hydrogen storage materials, as it directly governs structural integrity, operational safety, and durability under repeated hydrogen absorption–desorption cycles. In particular, hydride-based perovskites must withstand volumetric expansion, contraction, and stress accumulation without undergoing mechanical failure. To this end, the mechanical behaviour of the cubic Li-based hydride perovskites LiBH_3_, LiCuH_3_, LiMgH_3_, and LiSiH_3_ was systematically investigated through density functional theory (DFT)–derived elastic constants computed using the *thermo_pw* module of Quantum ESPRESSO.

Elastic properties provide a quantitative description of a material’s resistance to external stress and deformation, thereby offering key insight into its mechanical robustness during hydrogen cycling. For cubic perovskite hydrides, crystal symmetry reduces the number of independent elastic constants (${C}_{ij}$) to three, namely ${C}_{11}$, ${C}_{12}$, and ${C}_{44}$. Mechanical stability is assessed using the Born stability criteria for cubic systems, expressed as [60]:

7 $${C}_{11}>0, {C}_{44}>0, {C}_{11}-{C}_{12}>0, {C}_{11}+2{C}_{12}>0, {C}_{12}<B<{C}_{11}$$

All investigated compounds satisfy these criteria, indicating that LiBH_3_, LiCuH_3_, LiMgH_3_, and LiSiH_3_ are mechanically stable in their cubic phases. Nevertheless, significant differences in their elastic responses suggest varying degrees of mechanical robustness, which are critical for hydrogen storage applications.

#### Elastic moduli and mechanical rigidity

The DFT-calculated elastic constants form the basis for evaluating the bulk modulus (B) and shear modulus (G), which characterize resistance to uniform compression and shear deformation, respectively. These moduli were estimated using the Voigt–Reuss–Hill (VRH) averaging scheme, where the Voigt approximation assumes uniform strain, the Reuss approximation assumes uniform stress, and the Hill average provides a reliable intermediate representation of realistic elastic behaviour [61, 62].

The Voigt and Reuss moduli are given by [63, 64]:

8 $${B}_{R}={B}_{V}=\frac{{C}_{11}+{2C}_{12}}{3}$$

9 $${G}_{R}=\frac{5({C}_{11}-{C}_{12}){C}_{44}}{3\left({C}_{11}-{C}_{12}\right)+4{C}_{44}}$$

10 $${G}_{V}=\frac{(3{C}_{44}+{C}_{11}-{C}_{12})}{5}$$

The Hill-averaged bulk and shear moduli are then obtained as [65, 66]:

11 $$B=\frac{{B}_{V}+{B}_{R}}{2}$$

12 $$G=\frac{{G}_{V}+{G}_{R}}{2}$$

The calculated bulk moduli indicate that LiCuH_3_ (62.96 GPa) exhibits the highest resistance to volume compression, exceeding those of LiSiH_3_ (45.88 GPa), LiMgH_3_ (39.13 GPa) and LiBH_3_ (36.057 GPa) perovskite hydrides. However, bulk modulus alone does not fully describe mechanical rigidity. The shear modulus provides a more sensitive measure of resistance to shape deformation. In this regard, LiCuH_3_ exhibits the highest shear modulus (41.69 GPa), followed by LiMgH_3_ (24.63 GPa), LiSiH_3_ (22.42 GPa), and LiBH_3_ (13.62 GPa). The extremely low shear modulus of LiBH_3_ indicates weak resistance to shear deformation, suggesting mechanical softness despite its relatively high bulk modulus. In contrast, LiCuH_3_ demonstrates superior mechanical rigidity, combining high resistance to both volumetric and shear deformation. Therefore, among the studied compounds, LiCuH_3_ is expected to exhibit the greatest mechanical robustness under operational conditions [67].

#### Young’s modulus and elastic stiffness

Young’s modulus (E) quantifies resistance to uniaxial deformation and serves as a direct measure of overall material stiffness. It is calculated from B and G using [68]:

13 $$E=\frac{27GB}{9B+3G}$$

As presented in Table 2, the calculated Young’s modulus of LiBH_3_, LiCuH_3_, LiMgH_3_, and LiSiH_3_ are 36.283 GPa, 102.43 GPa, 61.08 GPa, and 57.85 GPa, respectively. These values confirm that LiCuH_3_ possesses the highest stiffness, followed by LiMgH_3_. The low Young’s modulus of LiBH_3_ further supports its classification as a mechanically soft material. Consequently, LiCuH_3_ is best suited to withstand repeated mechanical loading during hydrogen absorption–desorption cycles, while LiMgH_3_ offers moderate stiffness with acceptable mechanical stability.

**Table 2:** The calculated elastic constants ${C}_{ij}$ (GPa), Voigt–Reuss–Hill moduli ${B}_{V}$, ${B}_{R}$, B, ${G}_{V}$, ${G}_{R}$, G, ${E}_{V}$, ${E}_{R}$, E, (GPa), Pugh’s modulus G/B, Pugh’s ratio B/G, Poisson’s ratio v, Cauchy pressure ${C}_{p}$ (GPa), anisotropy ratio A, Ranganathan-Anisotropy Index ${A}^{U}$, Kleinman’s parameter ξ, micro-hardness H (GPa), Teter Vicker’s hardness ${H}^{TV}$(GPa), Chen, Vicker’s hardness ${H}^{CV}$(GPa) for LiBH_3_, LiCuH_3_, LiMgH_3_, and LiSiH_3_ hydride perovskites

Parameters	LiBH_3_	LiCuH_3_	LiMgH_3_	LiSiH_3_
${C}_{11}$	74.382	138.91	76.867	85.795
${C}_{12}$	16.895	24.981	20.262	25.918
${C}_{44}$	7.8265	33.758	22.450	18.455
${B}_{V}$	36.057	62.957	39.130	45.877
${B}_{R}$	36.057	62.957	39.130	45.877
B	36.057	62.957	39.130	45.877
${G}_{V}$	16.193	43.041	24.791	23.049
${G}_{R}$	11.04	40.330	24.475	21.8
G	13.62	41.685	24.633	22.424
${E}_{V}$	42.254	105.16	61.406	59.227
${E}_{R}$	30.053	99.701	60.757	56.457
E	36.283	102.43	61.081	57.848
B/G	2.65	1.50	1.59	2.05
G/B	0.131	0.66	0.63	0.49
${v}_{V}$	0.30469	0.22162	0.23846	0.28483
${v}_{R}$	0.36109	0.23606	0.24122	0.2949
v	0.33229	0.22860	0.23983	0.28984
${C}_{p}$	9.069	−8.777	−2.188	7.46
A	0.89	0.593	0.793	0.61
${A}^{U}$	0.02	0.34	0.07	0.29
ξ	0.522	0.331	0.413	0.449
${\mu}_{M}$	2.60	1.86	1.74	2.49
H	1.22	7.54	4.27	3.75
${H}^{TV}$	1.76	6.29	3.72	3.39
${H}^{CV}$	-	7.94	4.58	3.38

#### Bonding nature, ductility, and brittleness

The shear-to-bulk modulus ratio (G/B), known as Pugh’s modulus, provides insight into the nature of chemical bonding [88]. Values around 0.6 are indicative of predominantly ionic bonding, while values approaching 1.1 suggest covalent character. LiCuH_3_ (0.66) and LiMgH_3_ (0.63) exhibit values close to 0.6, indicating predominantly ionic bonding. LiSiH_3_ (0.49) shows a mixed ionic–metallic character, while the very low value for LiBH_3_ (0.13) suggests highly compliant bonding with weak shear resistance. The inverse ratio, B/G, known as Pugh’s ratio, distinguishes ductile from brittle behavior. Materials with B/G>1.75 are considered ductile, whereas lower values indicate brittleness [69]. LiCuH_3_ (1.50) and LiMgH_3_ (1.59) fall below the critical value of 1.75, indicating brittle behaviour. In contrast, LiSiH_3_ (2.05) and LiBH_3_ (2.65) lie within the ductile regime, although the latter reflects mechanical softness rather than true ductility.

Poisson’s ratio (v) further complements this analysis and is calculated as [70]:

14 $$v=\frac{3B-2G}{2(3B+G)}$$

Poisson’s ratios around 0.25 indicate ionic bonding, while values below 0.26 correspond to brittle behavior [71, 72]. LiCuH_3_ (0.229) and LiMgH_3_ (0.240) exhibit values consistent with ionic bonding and brittle behaviour, while LiSiH_3_ (0.290) and LiBH_3_ (0.332) show higher values indicative of increased ductility and reduced directional bonding strength.

#### Cauchy pressure and elastic anisotropy

Cauchy pressure (${C}_{p}$) provides further insight into ductility and bonding characteristics [27, 62]:

15 $${C}_{p}={C}_{12}-{C}_{44}$$

LiCuH_3_ (− 8.78 GPa) and LiMgH_3_ (− 2.19 GPa) exhibit negative values, confirming directional (angular) bonding and brittle character. Conversely, LiBH_3_ (9.07 GPa) and LiSiH_3_ (7.46 GPa) display positive values, consistent with metallic or ionic bonding and enhanced ductility.

Elastic anisotropy plays a crucial role in phonon transport, fracture behaviour, and deformation mechanisms. The shear anisotropy factor for cubic crystals is defined as:

16 $$A=\frac{2{C}_{44}}{{C}_{11}-{C}_{12}}$$

Ranganathan-Anisotropy Index (${A}^{U}$) for cubic crystals can be calculated by

17 $${A}^{U}=5\left(\frac{{G}_{V}-{G}_{R}}{{G}_{R}}\right)$$

**Fig. 7:**
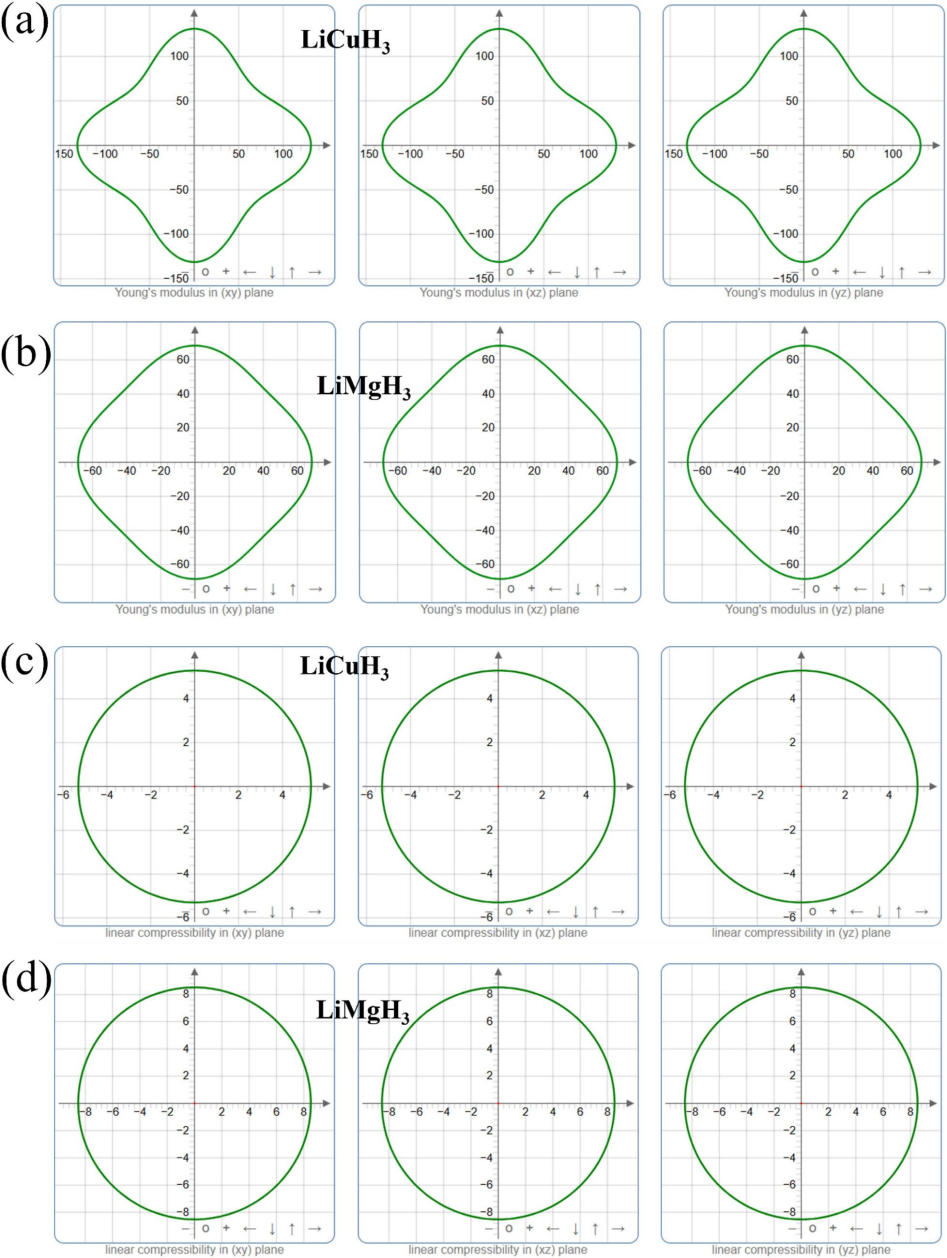
The 2D directional change of (**a**) Young’s modulus for LiCuH_3_, (**b**) Young’s modulus for LiMgH_3_, (**c**) linear compressibility for LiCuH_3_ and (**d**) linear compressibility for LiCuH_3_

**Fig. 8:**
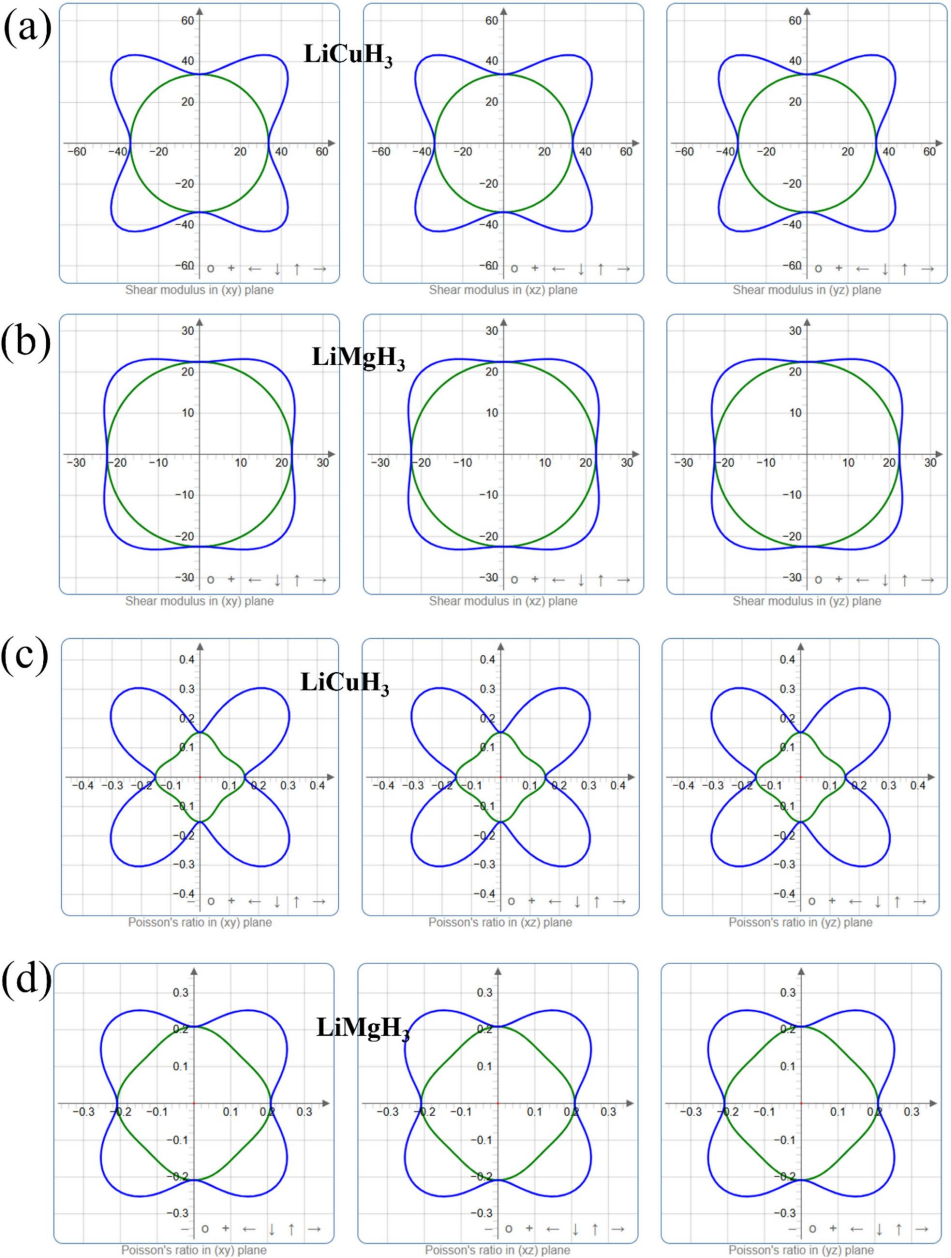
The 2D directional change of (**a**) Shear modulus for LiCuH_3_, (**b**) shear modulus for LiMgH_3_, (**c**) Poisson’s ratio for LiCuH_3_ and (**d**) Poisson’s ratio for LiCuH_3_

**Fig. 9:**
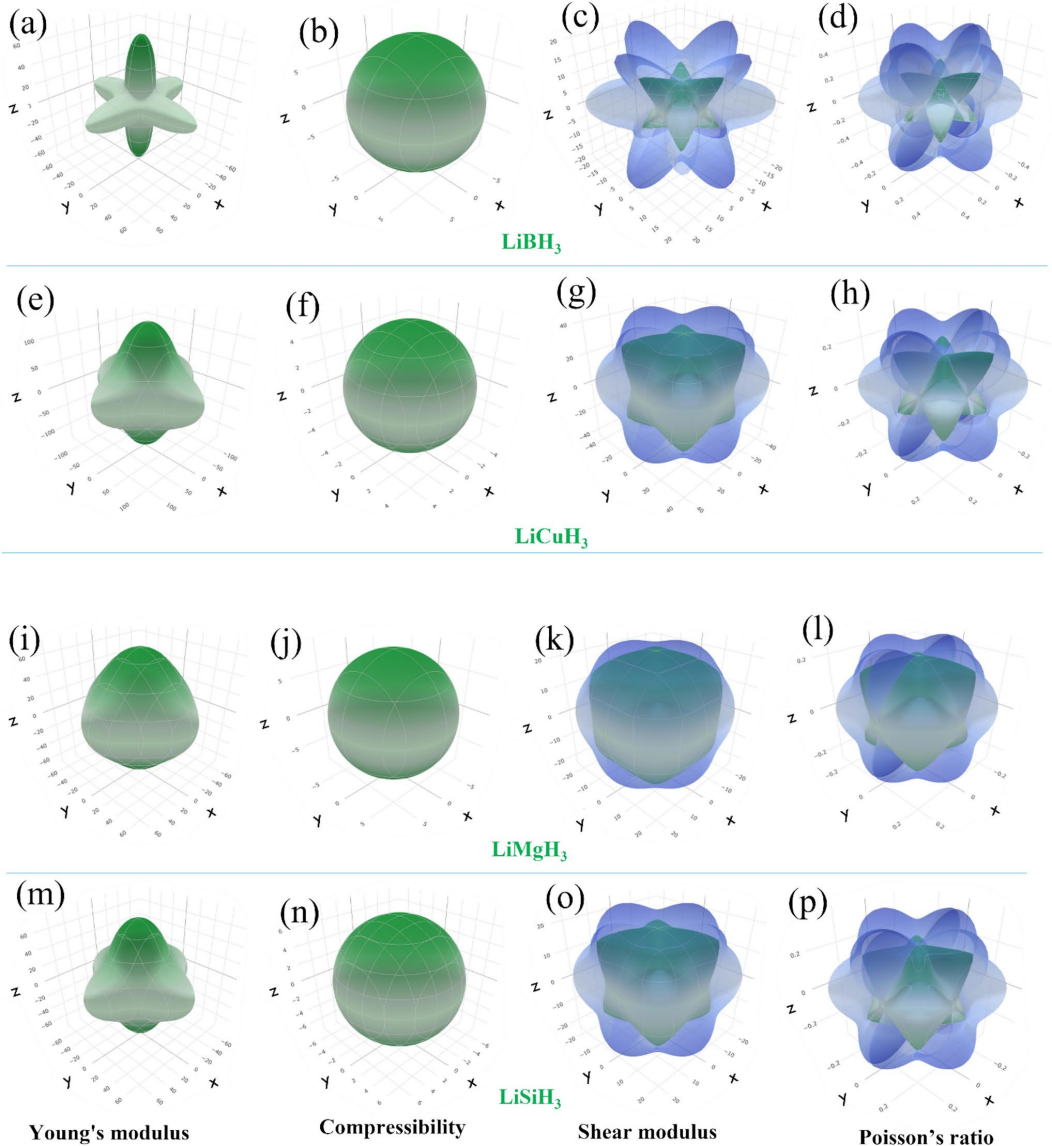
The 3D directional change of (**a**, **e**, **i**, **m**) Youngs modulus, (**b**, **f**, **j**, **n**) compressibility, (**c**, **g**, **k**, **o**) shear modulus and (**d**, **h**, **l**, **p**) Poisson’s ratio for LiBH_3_, LiCuH_3_, LiMgH_3_ and LiSiH_3_ perovskite hydrides

LiCuH_3_ and LiMgH_3_ exhibit moderate anisotropy, while LiBH_3_ and LiSiH_3_ show pronounced anisotropic behaviour due to negative shear elastic constants, reflecting mechanical instability. To further elucidate directional elastic behaviour, 2D and 3D elastic property visualizations were generated using the ELATE software [73]. As illustrated in Figs. 7, 8 and 9, the studied perovskite hydrides display nearly isotropic linear compressibility, whereas Young’s modulus, shear modulus, and Poisson’s ratio exhibit anisotropic characteristics.

#### Kleinman’s parameter, machinability, and hardness

Kleinman’s parameter (ξ) evaluates the relative contributions of bond stretching and bond bending [53]:

18 $$\xi =\frac{{C}_{11}+8{C}_{12}}{7{C}_{11}+2{C}_{12}}$$

LiCuH_3_ (0.331) and LiMgH_3_ (0.413) show lower values, suggesting dominance of bond bending, whereas LiBH_3_ (0.522) indicates a more balanced deformation mechanism.

Machinability index (${\mu}_{M}$), crucial for large-scale fabrication, is assessed via the machinability index [36]:

19 $${\mu}_{M}=\frac{B}{{C}_{44}}$$

The machinability index reveals that LiBH_3_ (2.60) and LiSiH_3_ (2.49) are more easily machinable, while LiCuH_3_ (1.86) and LiMgH_3_ (1.74) exhibit moderate machinability, consistent with their higher stiffness.

The micro-hardness (H) of a material can be estimated using its elastic properties. It is determined from the following relation [18]:

20 $$H=\frac{E\left(1-2v\right)}{6\left(1+v\right)}$$

where E is Young’s modulus and ν is Poisson’s ratio. This equation links hardness to the elastic response of the material, providing insight into its resistance to localized deformation. LiCuH_3_ emerges as the hardest mechanically stable compound, followed by LiMgH_3_ presented in Table 2. Teter and Chen hardness models were applied only where physically meaningful (G>0):

21 $${H}^{TV}=0.151G$$

22 $${H}^{CV}=2{\left(\frac{G}{{k}^{2}}\right)}^{0.585}-3$$

where k is Pugh’s ratio. As presented in Table 2 Teter and Chen hardness models result shows that LiCuH_3_ is harder compared to LiMgH_3_.

### Thermodynamic properties

The thermodynamic performance of hydrogen storage materials governs their operational temperature window, reversibility, and energetic efficiency. For Li-based hydride perovskites LiXH_3_ (X = B, Cu, Mg, Si), thermodynamic properties were systematically evaluated using ab initio density functional theory combined with the quasi-harmonic approximation (QHA), as implemented in the thermo_pw module of *Quantum ESPRESSO*. This framework enables reliable prediction of temperature-dependent enthalpy, Gibbs free energy, entropy, heat capacity, and lattice vibrational characteristics key quantities for assessing hydrogen adsorption–desorption behaviour under realistic operating conditions.

#### Enthalpy and Gibbs free energy

**Fig. 10:**
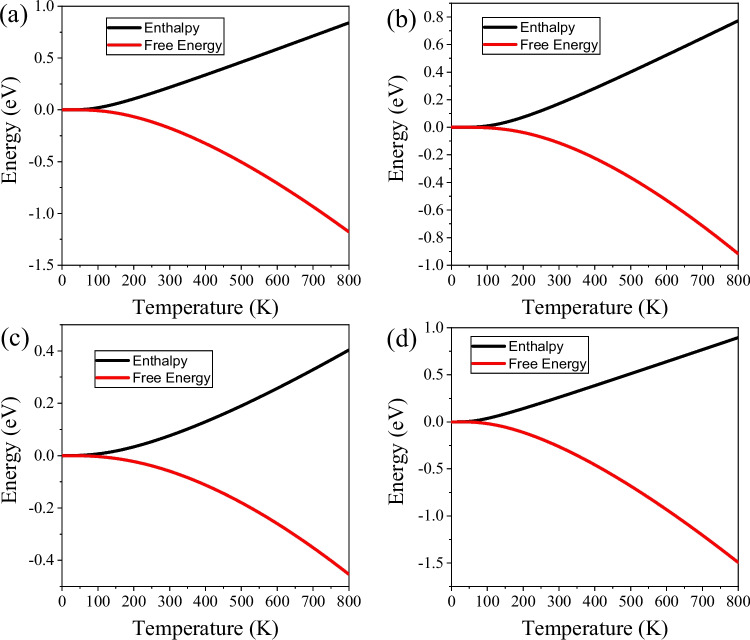
Enthalpy and free energy of (**a**) LiBH_3_, (**b**) LiCuH_3_, (**c**) LiMgH_3_, and (**d**) LiBH_3_ perovskite hydrides

Enthalpy (ΔH) reflects the heat absorbed or released during hydrogen storage processes and provides direct insight into metal–hydrogen bond strength. Higher enthalpy values generally indicate stronger bonding and improved thermal stability, whereas lower enthalpy favors facile hydrogen release and reversibility [74]. As illustrated in Fig. 10(a–d), the enthalpy of all LiXH_3_ hydride perovskites increases nearly linearly with temperature, reflecting progressive vibrational energy accumulation within the lattice. Notably, LiBH_3_ and LiSiH_3_ exhibit steeper enthalpy slopes compared with LiCuH_3_ and LiMgH_3_, indicating enhanced thermal responsiveness and greater vibrational energy storage per unit temperature rise. This behavior suggests stronger phonon–lattice coupling and higher internal energy retention in LiBH_3_ and LiSiH_3_, consistent with their stiffer vibrational modes. In contrast, the more moderate enthalpy evolution observed for LiCuH_3_ and LiMgH_3_ is advantageous for reversible hydrogen cycling, as it balances thermal stability with manageable desorption energetics.

The Gibbs free energy (ΔG) governs the thermodynamic spontaneity of hydrogen desorption. For spontaneous hydrogen release, ΔG must be negative, and a monotonic decrease with temperature indicates enhanced favorability at elevated conditions [18, 62]. As shown in Fig. 10(a–d), ΔG decreases steadily with increasing temperature for all studied hydride perovskites, confirming that hydrogen desorption becomes thermodynamically favorable at higher temperatures. Among the investigated systems, LiSiH_3_ exhibits the most negative Gibbs free energy (≈ − 1.5 eV), followed by LiBH_3_ (≈ − 1.2 eV), LiCuH_3_ (≈ − 0.9 eV), and LiMgH_3_ (≈ − 0.4 eV). These trends indicate stronger thermodynamic driving forces for phase stability and transformation in LiSiH_3_ and LiBH_3_. Importantly, the consistently negative free energy values across all compositions demonstrate that LiXH_3_ (X = B, Cu, Mg, Si) hydride perovskites remain thermodynamically stable over the investigated temperature range, supporting their suitability for high-temperature hydrogen storage applications.

#### Entropy and heat capacity

**Fig. 11:**
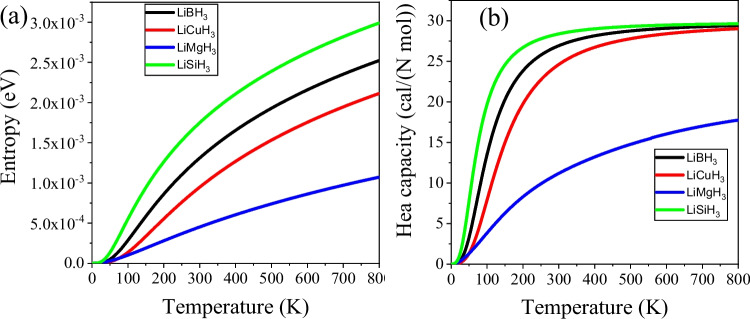
(**a**) Entropy and (**b**) heat capacity for LiBH_3_, LiCuH_3_, LiMgH_3_, and LiSiH_3_, perovskite hydrides

Entropy (S) measures the degree of disorder and configurational freedom in a system and plays a critical role in hydrogen desorption kinetics. As shown in Fig. 11a, the entropy of LiBH_3_, LiCuH_3_, LiMgH_3_, and LiSiH_3_ increases monotonically with temperature. This entropy enhancement lowers the free-energy barrier for hydrogen release, thereby promoting desorption and improving kinetic performance at elevated temperatures.

The temperature dependence of the constant-volume heat capacity (${C}_{V}$) is presented in Fig. 11b. At low temperatures (T≲300 K), ${C}_{V}$​ rises sharply with temperature due to the activation of low-frequency phonon modes, following the Debye ${C}_{V}\propto {T}^{3}$ law. As temperature increases further, ${C}_{V}$​ gradually approaches the classical Dulong–Petit limit (${C}_{V}=3n{N}_{A}{k}_{B}$​), indicating saturation of vibrational degrees of freedom and classical lattice behaviour. Among the studied compounds, LiMgH_3_ exhibits the lowest heat capacity, implying reduced vibrational energy storage and potentially faster thermal equilibration. This characteristic is beneficial for applications requiring rapid heat dissipation during hydrogen cycling.

#### Zero-point energy and vibrational stability

Zero-point energy (ZPE), which originates from quantum mechanical lattice vibrations persisting even at absolute zero temperature, is an essential contribution to the total energy of solids and plays a critical role in accurately evaluating the thermodynamic stability of hydrogen-rich materials [75]. In hydride systems, ZPE corrections are particularly important due to the low mass of hydrogen, which leads to significant vibrational contributions. Generally, higher ZPE values reflect increased intrinsic lattice energy and may slightly reduce the overall stability of the hydride framework.

The calculated ZPE values for LiBH_3_, LiCuH_3_, LiMgH_3_, and LiSiH_3_ are 0.21 eV, 0.24 eV, 0.30 eV, and 0.15 eV, respectively. Among the investigated compounds, LiSiH_3_ exhibits the lowest ZPE, indicating a reduced baseline vibrational energy and suggesting comparatively enhanced intrinsic lattice stability. In contrast, LiMgH_3_ shows the highest ZPE, implying stronger vibrational contributions that may marginally destabilize the lattice. From a hydrogen storage perspective, lower ZPE values are advantageous, as they reduce the vibrational energy contribution to the system and consequently lower the energetic penalty associated with hydrogen adsorption and desorption processes. This can facilitate more favorable thermodynamics for hydrogen release and uptake, thereby improving storage efficiency. Furthermore, when compared with previously reported perovskite hydrides such as MgFeH_3_ (ZPE ≈ 0.6 eV) [76], the hydrides investigated in this study exhibit significantly lower zero-point energies. This substantial reduction in vibrational energy highlights the improved thermodynamic favorability of the present Li-based hydride perovskites and reinforces their potential as efficient hydrogen storage materials.

#### Debye temperature and melting temperature

The Debye temperature (${\Theta}_{D}$​) provides valuable insight into lattice stiffness, phonon spectra, specific heat behaviour, and thermal conductivity [77]. It establishes a direct link between elastic and vibrational properties via the average sound velocity (${v}_{m}$​), derived from longitudinal (${v}_{l}$) and transverse (${v}_{t}$​) sound velocities:

23 $${v}_{m}={\left[\frac{1}{3}\left(\frac{2}{{v}_{l}^{3}}+\frac{1}{{v}_{t}^{3}}\right)\right]}^{-\frac{1}{3}}$$

where

24 $${v}_{l}=\sqrt{\frac{3B+4G}{\rho }},{ v}_{t}=\sqrt{\frac{G}{\rho }}$$

and

25 $${\Theta}_{D}=\frac{h}{{k}_{B}}{\left(\frac{3n{N}_{A}\rho }{4\pi M}\right)}^{{~}^{1}\!\left/ \!{~}_{3}\right.}{v}_{m}$$

Here, h is Planck’s constant, ​${k}_{B}$ is Boltzmann’s constant, ${N}_{A}$​ is Avogadro’s number, n is the number of atoms per formula unit, M is the molecular mass, and ρ is the density.

Among the studied materials, LiMgH_3_ exhibits the highest Debye temperature (718.94 K), indicating strong interatomic bonding, enhanced lattice rigidity, and high resistance to thermal vibrations. LiCuH_3_ also shows a high ${\Theta}_{D}$ value (600.88 K), reflecting robust bonding strength and good thermal stability, which are essential for maintaining structural integrity during repeated hydrogen absorption–desorption cycles. LiSiH_3_ presents an intermediate Debye temperature (520.22 K), suggesting moderate lattice stiffness and balanced vibrational stability. In contrast, LiBH_3_ exhibits the lowest Debye temperature (345 K) among the studied compounds, indicating comparatively weaker lattice rigidity and stronger phonon anharmonicity. This suggests higher vibrational freedom and lower resistance to thermal perturbations, although LiBH_3_ remains mechanically stable within the elastic stability framework.

Thermal resilience is further assessed through the melting temperature (${T}_{m}$), which correlates with the strength of interatomic interactions [78]. The melting temperature is estimated using [53]:

26 $${T}_{m}=\left[553\left(K\right)+5.911{C}_{12}\right]GPa\pm 300K$$

As summarized in Table 3, the calculated melting temperatures indicate that LiSiH_3_ exhibits the highest thermal resistance (706.4 K) among the investigated systems, followed closely by LiCuH_3_ (700.6 K) and LiMgH_3_ (672.8 K). These values are consistent with their relatively high Debye temperatures and mechanically stable frameworks. LiBH_3_ shows a moderate melting temperature (651.6 K), which is lower than that of LiCuH_3_ LiSiH_3_, and LiMgH_3_ reflecting its comparatively weaker bonding strength and reduced lattice rigidity.

**Table 3:** The DFT calculated ${v}_{l}$, ${v}_{t}$, ${v}_{m}$, ${\Theta}_{D}$ and ${T}_{m}$ of various hydride perovskites

Hydrides	${v}_{l}$ (km/s)	${v}_{t}$(km/s)	${v}_{m}$(km/s)	${\Theta}_{D}$ (K)	${T}_{m}$(K)	Ref
LiBH_3_	4.20	2.20	2.45	345	651.6	This work
LiCuH_3_	6.515	3.616	4.010	600.878	700.6	This work
LiMgH_3_	7.560	4.792	5.250	718.942	672.8	This work
LiSiH_3_	3.51	6.89	3.93	520.217	706.4	This work
MgSiH_3_	7.4551	4.3549	5.0557	154.98	567.42	[18]
NaSiH_3_	7.0343	4.1402	4.7983	145.85	669.17	[18]
KSiH_3_	5.1732	2.8907	3.3892	184.40	627.83	[18]
LiSiH_3_	9.1059	5.4276	6.2721	198.42	756.26	[18]
MgCrH_3_	−	−	5.032	756.52	1936	[79]
CaCrH_3_	−	−	4.781	678.45	1670	[79]
MgAlH_3_	6.229	2.786	3.143	427.539	−	[80]
NaZrH_3_	5.710	3.252	3.590	459.702	1247.975	[81]
NaScH_3_	6.733	4.046	4.445	570.82	1146.151	[81]
MgRuH_3_	4.761	2.826	3.008	438.45	966.11	[82]
BaReH_3_	2.683	2.324	2.568	331.62	862.73	[82]

### Electronic properties

#### Band structure

**Fig. 12:**
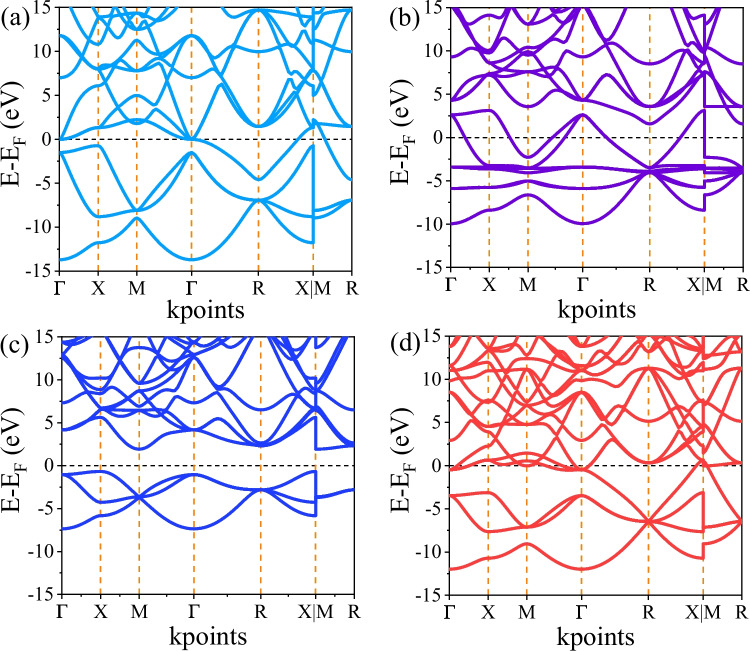
The computed band structure for (**a**) LiBH_3_, (**b**) LiCuH_3_, (**c**) LiMgH_3_, and (**d**) LiSiH_3_ perovskite hydrides

The electronic band structure describes the allowed energy states of electrons in a crystalline solid and provides fundamental insight into its conductive nature. The energy separation between the valence band (VB) and conduction band (CB), known as the bandgap, determines whether a material behaves as a metal, semiconductor, or insulator. The calculated band structures of LiBH_3_, LiCuH_3_, LiMgH_3_, and LiSiH_3_ along high-symmetry directions in the Brillouin zone are presented in Fig. 12(a–d). The Fermi level is set to 0 eV and indicated by a horizontal dashed line.

As shown in Fig. 12, LiBH_3_, LiCuH_3_, and LiSiH_3_ exhibit metallic behavior, characterized by a pronounced overlap between the valence and conduction bands at the Fermi level. Such metallicity has been widely reported for several perovskite hydrides in the literature and is highly advantageous for hydrogen storage applications [83]. The presence of delocalized conduction electrons enhances electrical conductivity and facilitates rapid charge transfer within the lattice. This conductive environment promotes hydrogen diffusion between interstitial sites and improves both absorption and desorption kinetics [62]. Moreover, electronic screening by mobile electrons can mitigate local lattice strain induced during hydrogen insertion and extraction, thereby contributing to improved mechanical resilience and cycling stability.

In contrast, LiMgH_3_ exhibits a semiconducting nature, with an indirect bandgap of approximately 2.59 eV, as shown in Fig. 12c. The conduction band minimum (CBM) is located at the M high-symmetry point, while the valence band maximum (VBM) occurs at the X point. The existence of a forbidden energy gap separates the VB and CB, restricting free electron availability at the Fermi level. Similar semiconducting behavior has been reported for several perovskite hydrides. For example, Ayyaz et al. [53] reported perovskite metal hydrides MgLiH_3_, CaLiH_3_, SrLiH_3_, and BaLiH_3_ with bandgap values of 2.66, 2.34, 1.94, and 1.37 eV, respectively. Song et al. [45] also reported bandgap of 1.85 eV and 2.83 eV for LiSrH_3_ and RbSrH_3_, respectively. While semiconducting hydrides may offer enhanced chemical stability, metallic hydride perovskites are generally more favorable for hydrogen storage due to their superior electronic conductivity and enhanced hydrogen mobility. Consequently, the metallic nature of LiBH_3_, LiCuH_3_, and LiSiH_3_ suggests a strong potential for fast hydrogen diffusion and improved storage–release kinetics, whereas LiMgH_3_ may require higher activation energies for hydrogen transport.

#### Density of states

**Fig. 13:**
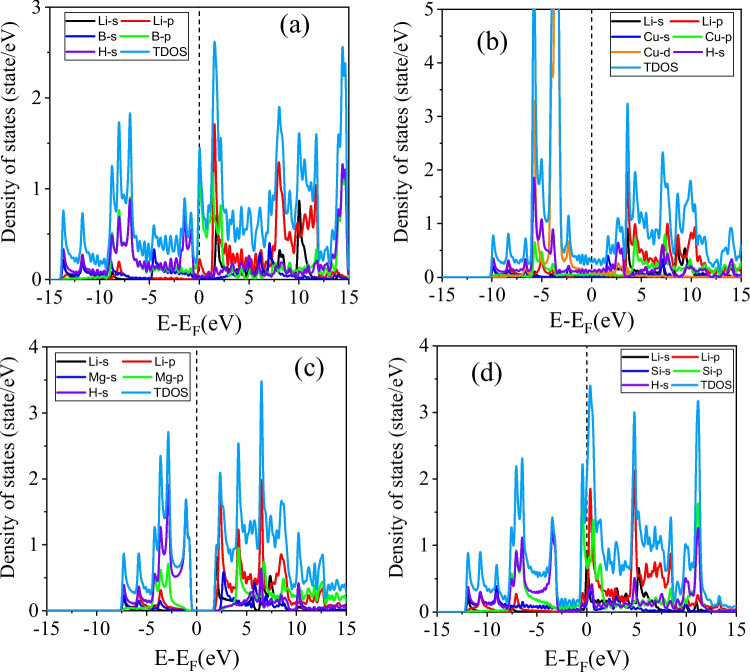
The computed PDOS and TDOS for (**a**) LiBH_3_, (**b**) LiCuH_3_, (**c**) LiMgH_3_, and (**d**) LiSiH_3_ perovskite hydrides

The electronic density of states (DOS) provides detailed insight into the distribution of available electronic energy levels and their orbital contributions. In particular, the projected density of states (PDOS) enables identification of the atomic orbitals responsible for the formation of the valence and conduction bands, thereby clarifying bonding characteristics and charge-transfer pathways. The calculated total density of states (TDOS) and PDOS for LiBH_3_, LiCuH_3_, LiMgH_3_, and LiSiH_3_ are shown in Fig. 13(a–d), with the Fermi level indicated by a vertical dotted line.

Consistent with the band structure analysis, LiBH_3_, LiCuH_3_, and LiSiH_3_ exhibit finite DOS at the Fermi level, confirming their metallic character, whereas LiMgH_3_ shows a clear bandgap around the Fermi level, indicative of semiconducting behaviour. For LiBH_3_ (Fig. 13a), the valence band below the Fermi level is predominantly composed of B-p, H–s, and B-s orbitals, with minor contributions from Li–s and Li-p states. The conduction band is mainly formed by B-p and Li-p orbitals, accompanied by smaller contributions from B-s and Li–s states. These orbital hybridizations give rise to pronounced TDOS peaks at − 13.62, − 11.71, − 8.76, − 8.06, − 6.94, and − 1.45 eV in the valence band, and at 0.04, 1.53, 8.02, 10.0, 11.75, and 14.36 eV in the conduction band. The strong hybridization between B and H states highlights robust metal–hydrogen bonding and electronic delocalization.

In LiCuH_3_ (Fig. 13b), the valence band is dominated by Cu-d orbitals, with minor contributions from H–s, Cu–s, Cu-p, and Li-p states. The conduction band is primarily composed of Li-p and Cu-p orbitals, with smaller contributions from H–s, Li–s, and Cu–s states. The prominent TDOS peaks appear at − 5.78 and − 3.50 eV in the valence band and at 3.60, 7.16, and 9.96 eV in the conduction band. The strong presence of Cu-d states near the Fermi level enhances electronic conductivity and is particularly favorable for charge-mediated hydrogen diffusion.

For LiMgH_3_ (Fig. 13c), the valence band is mainly formed by H–s and Mg-p orbitals, with minor Li–s and Li-p contributions. The conduction band consists predominantly of Li-p and Mg-p states, accompanied by smaller contributions from Li–s, H–s, and Mg-s orbitals. The absence of states at the Fermi level confirms its semiconducting nature and explains the reduced electronic conductivity compared to the metallic hydrides. In LiSiH_3_ (Fig. 13d), the valence band arises from strong hybridization among H–s, Si-p, Li-p, and Si-s orbitals, while the conduction band is dominated by H–s, Si-p, Li-p, and Li–s states. The finite DOS at the Fermi level confirms metallic behaviour and suggests efficient charge transfer pathways that can facilitate hydrogen mobility.

#### Bader charge analysis

To quantitatively evaluate electron redistribution in LiXH_3_ (X = B, Cu, Mg, Si) perovskite hydrides, we performed Bader charge analysis, with the results summarized in Table 4. The calculated partial net charges reveal a clear trend in charge transfer and bonding character across the series. Positive Bader charges indicate electron donation, while negative values correspond to electron gain. In all compounds, Li atoms donate a significant amount of charge (+ 1.44 to + 1.56 e), confirming their role as nearly fully ionized Li^+^ cations, consistent with the highly electropositive nature of lithium. Hydrogen atoms, on the other hand, carry substantial negative charges ranging from − 2.11 to − 2.97 e (total over three H atoms), indicating their anionic character (H⁻) and confirming the hydride nature of these materials.

**Table 4:** Bader partial net charges for LiXH_3_ (X = B, Cu, Mg, Si) perovskite hydrides

Hydrides	Li	B	Cu	Mg	Si	H
LiBH_3_	+ 1.48	+ 0.63	−	−	−	− 2.11
LiCuH_3_	+ 1.51	−	+ 0.95	−	−	− 2.46
LiMgH_3_	+ 1.56	−	−	+ 1.41	−	− 2.97
LiSiH_3_	+ 1.44	−	−	−	+ 0.79	− 2.23

A notable variation is observed in the charge states of the X-site cations. LiMgH_3_ exhibits the largest positive charge on Mg (+ 1.41 e) and the most negative total charge on hydrogen (− 2.97 e). This indicates strong charge transfer from Mg to H, reflecting highly ionic Mg–H interactions. Such pronounced ionicity is consistent with the low electronegativity of Mg and explains the localized electronic states and semiconducting behavior observed in this compound. In contrast, LiBH_3_ (+ 0.63 e on B) and LiSiH_3_ (+ 0.79 e on Si) show significantly smaller charge transfer to hydrogen. This reduced ionicity suggests stronger covalent character in B–H and Si–H bonds, arising from effective orbital hybridization between H 1 s and B/Si p states. The partial covalency leads to enhanced electronic delocalization, which contributes to their metallic features. LiCuH_3_ presents an intermediate case, with Cu carrying a charge of + 0.95 e. Although the charge transfer is larger than in B and Si systems, it remains lower than in Mg-based hydride. The involvement of Cu 3d states near the Fermi level likely enhances hybridization with H 1 s orbitals, giving rise to mixed ionic–covalent bonding and metallic conductivity.

Importantly, the magnitude of negative charge on hydrogen provides insight into hydrogen binding strength. The highly negative hydrogen in LiMgH_3_ suggests stronger electrostatic interaction and higher hydrogen binding energy, which may improve thermodynamic stability but hinder hydrogen desorption. Conversely, the relatively less negative hydrogen in LiBH_3_ and LiSiH_3_ indicates weaker binding, which could facilitate hydrogen release and improve storage kinetics.

#### Electron localization function

**Fig. 14:**
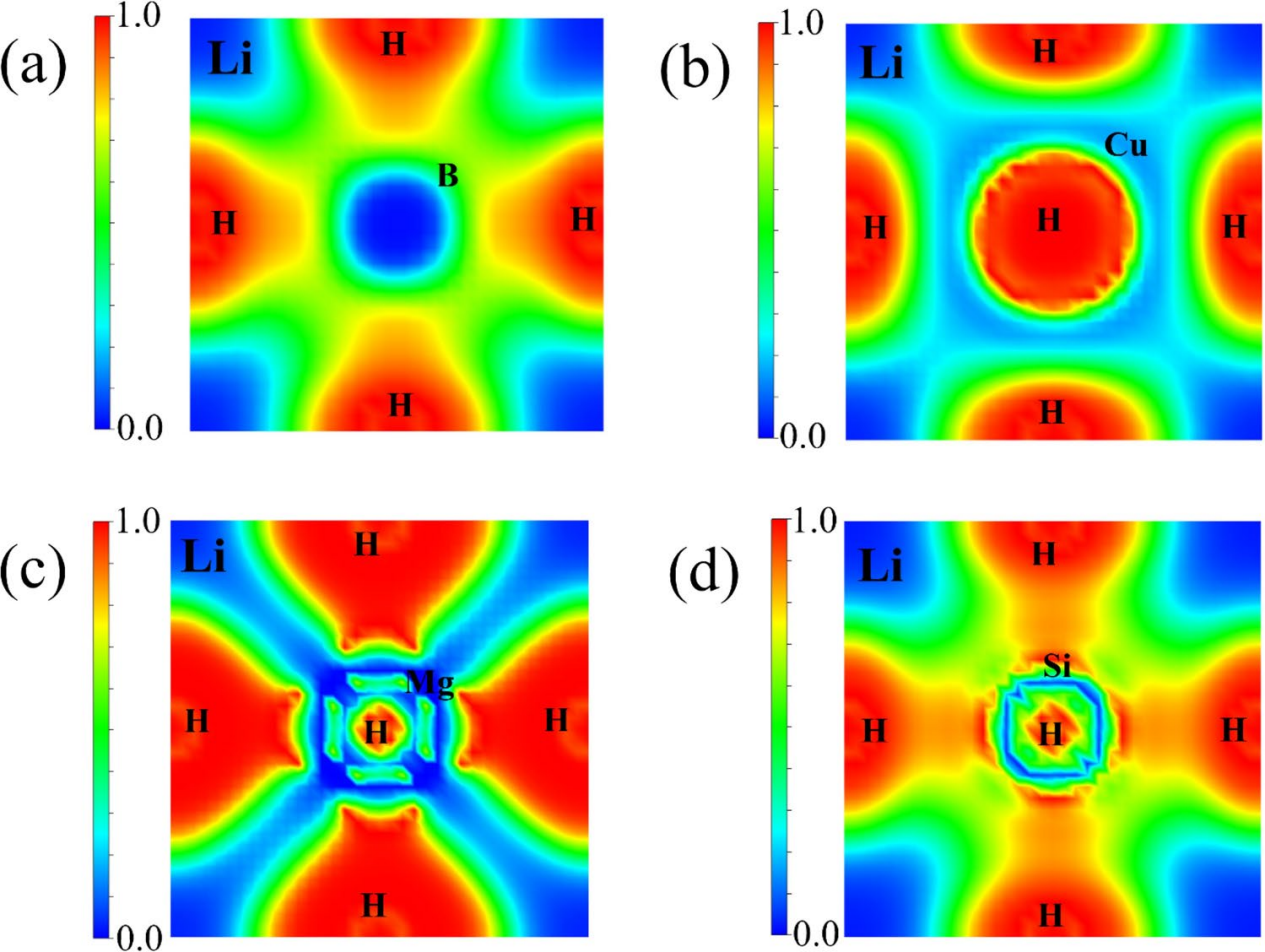
The ELF for (**a**) LiBH_3_, (**b**) LiCuH_3_, (**c**) LiMgH_3_, and (**d**) LiSiH_3_ perovskite hydrides

The electron localization function (ELF) is a powerful tool for visualizing the spatial distribution of electrons and assessing the nature of chemical bonding in solids. ELF values range from 0 to 1, where values close to 1 indicate highly localized electrons (typically associated with covalent bonding or lone pairs), whereas values approaching 0 correspond to delocalized, metallic-like electron behavior [84]. An ELF value of 0.5 represents a homogeneous electron gas distribution. The calculated ELF maps for LiBH_3_, LiCuH_3_, LiMgH_3_, and LiSiH_3_ are presented in Fig. 14(a–d), providing important insights into the bonding characteristics of these perovskite hydrides.

In LiBH_3_ (Fig. 14a), strong electron localization (ELF ≈ 1) is observed around the hydrogen atoms, confirming that hydrogen acts as an electron acceptor, consistent with its hydride (H⁻) character. Around the B atom, a moderate ELF distribution is observed, indicating noticeable B–H orbital hybridization. This suggests that, although charge is transferred toward hydrogen, the B–H bonds possess a mixed ionic–covalent character, rather than being purely ionic. This observation is in good agreement with the relatively smaller Bader charge on B (+ 0.63 e), which reflects limited charge transfer and enhanced covalency.

For LiCuH_3_ (Fig. 14b), the ELF map shows pronounced localization around hydrogen atoms, while the region around Cu exhibits comparatively lower but non-negligible electron density with slight anisotropy. This indicates that electrons are transferred from Li and Cu to hydrogen; however, the presence of finite electron density around Cu suggests hybridization between Cu 3d states and H 1 s orbitals. Therefore, the Cu–H bonding can be described as mixed ionic–covalent, rather than purely ionic. Importantly, there is no clear evidence of direct Li–Cu covalent bonding; instead, Li primarily acts as an electron donor, consistent with its high positive Bader charge (+ 1.51 e).

In contrast, LiMgH_3_ (Fig. 14c) exhibits highly localized electron density almost exclusively around hydrogen atoms, with very low ELF values in the interstitial region between Mg and H. This indicates minimal orbital overlap and confirms that the Mg–H interaction is predominantly ionic in nature. This is further supported by the largest Bader charge on Mg (+ 1.41 e) and the most negative hydrogen charge (− 2.97 e), reflecting significant charge transfer. The absence of directional electron localization between Mg and H is consistent with weak covalency and explains the semiconducting behavior observed in this compound. In LiSiH_3_ (Fig. 14d), strong electron localization is again observed around hydrogen atoms, but unlike LiMgH_3_, there is noticeable electron density surrounding the Si atom and along the Si–H bonding direction. This indicates significant orbital hybridization between Si 3p and H 1 s states, resulting in mixed ionic–covalent bonding. The intermediate Bader charge on Si (+ 0.79 e) further supports this interpretation, placing LiSiH_3_ between the strongly ionic LiMgH_3_ and more covalent LiBH_3_ systems.

### Optical properties

The optical properties of perovskite hydrides play a critical role in elucidating their interaction with electromagnetic radiation and provide indirect yet valuable insight into their electronic structure, bonding characteristics, and polarization behaviour. These features are intimately linked to hydrogen adsorption–desorption mechanisms, lattice polarizability, and charge redistribution, all of which are central to the performance of solid-state hydrogen storage materials [85]. In this context, a comprehensive analysis of the dielectric response, refractive index, absorption, reflectivity, energy-loss function, and optical conductivity was carried out for LiBH_3_, LiCuH_3_, LiMgH_3_, and LiSiH_3_ perovskite hydrides.

The frequency-dependent optical response of a material is described by the complex dielectric function:

27 $$\varepsilon \left(\omega \right)={\varepsilon}_{1}\left(\omega \right)+i{\varepsilon}_{2}(\omega )$$

In this equation ${\varepsilon}_{1}\left(\omega \right)$ is real part of dielectric function which can be calculated by Kramers–Kronig relation [52].

28 $${\varepsilon}_{1}\left(\omega \right)=1+\frac{2}{\pi }P{\int}_{0}^{\infty }\frac{{\varepsilon}_{2}{\omega }{\prime}({\omega }{\prime})}{{\omega }^{{\prime}2}-{\omega }^{2}}d\omega$$

where P Cauchy principal value of the integral.

The real part, ${\varepsilon}_{1}\left(\omega \right)$, represents the dispersive response and polarization of the medium under an external electromagnetic field and is obtained through the Kramers–Kronig transformation. In contrast, the imaginary part, ${\varepsilon}_{2}(\omega )$, arises from direct interband electronic transitions between occupied valence states and unoccupied conduction states and governs the absorption characteristics of the material.

29 $${\varepsilon}_{2}(\omega )=\frac{8{\pi }^{2}{e}^{2}}{{\omega }^{2}{m}^{2}}\sum_{n}\sum_{{n}{\prime}}{\left|{P}_{n{n}{\prime}}^{v}\right|}^{2}{f}_{kn}\left(1-{f}_{n{n}{\prime}}\delta \left({E}_{n}^{k}-{E}_{{n}{\prime}}^{k}-\omega \right)\right)\frac{{d}^{3}k}{{\left(2\pi \right)}^{3}}$$

where n and ${n}{\prime}$ denotes occupied (valence) states and unoccupied (conduction) states, ${P}_{n{n}{\prime}}^{v}$ is matrix element of the momentum operator, ${f}_{kn}$ and ${f}_{n{n}{\prime}}$ are Fermi–Dirac occupation functions for the initial and final states at wave vector, and ${E}_{n}^{k}$ and ${E}_{{n}{\prime}}^{k}$ energies of the electronic states.

**Fig. 15:**
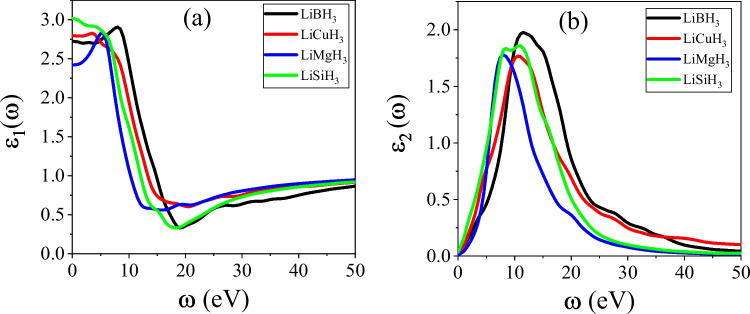
The computed (**a**) real part and (**b**) imaginary part dielectric function for LiBH_3_, LiCuH_3_, LiMgH_3_, and LiSiH_3_ perovskite hydrides

Figures 15a and b present the calculated real and imaginary components of the dielectric function for the studied perovskite hydrides over the photon energy range of 0–50 eV. The static dielectric constants ${\varepsilon}_{1}\left(0\right)$ are found to be 2.73, 2.80, 2.42, and 3.02 for LiBH_3_, LiCuH_3_, LiMgH_3_, and LiSiH_3_, respectively. In the low-energy region (0–4.5 eV), ${\varepsilon}_{1}\left(\omega \right)$, increases in the order LiMgH_3_ < LiBH_3_ < LiCuH_3_ < LiSiH_3_, indicating that LiSiH_3_ exhibits the highest electronic polarizability among the investigated compounds, followed closely by LiCuH_3_. High dielectric polarization is desirable for hydrogen storage materials, as it enhances the interaction between hydrogen species and the host lattice, facilitating reversible hydrogen uptake and release [35, 43]. From this perspective, LiSiH_3_ and LiCuH_3_ emerge as promising candidates due to their superior polarization response.

The imaginary part ${\varepsilon}_{2}\left(\omega \right)$, shown in Fig. 15b, reveals pronounced absorption peaks associated with interband electronic transitions. The main peaks are located at approximately 11.5 eV for LiBH_3_, 10.5 eV for LiCuH_3_, 8.0 eV for LiMgH_3_, and 11.0 eV for LiSiH_3_. These features reflect differences in band structure and bonding strength, with higher ${\varepsilon}_{2}\left(\omega \right)$ values indicating stronger photon absorption. Materials exhibiting large ${\varepsilon}_{2}\left(\omega \right)$ are often considered favorable for hydrogen storage applications, as enhanced electronic transitions can support photon-assisted processes and energy transfer mechanisms relevant to hydrogen sorption dynamics [86].

From real and imaginary part of the dielectric functions, other important optical properties such as refractive index $n\left(\omega \right)$*,* extinction coefficient $k\left(\omega \right)$*,* absorption coefficient $\alpha \left(\omega \right)$*,* reflectivity R(ω)*,* conductivity σ(ω), and electron energy loss spectrum $L\left(\omega \right)$ can be derived [87–89].

30 $$n\left(\omega \right)=\frac{1}{\sqrt{2}}{\left(\sqrt{{\varepsilon}_{1}^{2}\left(\omega \right)+{\varepsilon}_{2}^{2}\left(\omega \right)}+{\varepsilon}_{1}(\omega )\right)}^{{~}^{1}\!\left/ \!{~}_{2}\right.}$$

31 $$k\left(\omega \right)=\frac{1}{\sqrt{2}}{\left(\sqrt{{\varepsilon}_{1}^{2}\left(\omega \right)+{\varepsilon}_{2}^{2}\left(\omega \right)}-{\varepsilon}_{1}(\omega )\right)}^{{~}^{1}\!\left/ \!{~}_{2}\right.}$$

32 $$\alpha \left(\omega \right)=\sqrt{2}\omega \left(\sqrt{{\varepsilon}_{1}\left(\omega \right)+{\varepsilon}_{2}(\omega )}-{\varepsilon}_{1}(\omega )\right)$$

33 $$R(\omega )=\frac{1+{n}^{2}-2n+{\kappa }^{2}}{1+{n}^{2}+2n+{\kappa }^{2}}$$

34 $$L\left(\omega \right)=\frac{{\varepsilon}_{2}(\omega )}{{\varepsilon}_{2}^{2}\left(\omega \right)+{\varepsilon}_{1}^{2}\left(\omega \right)}$$

35 $$\sigma \left(\omega \right)=\frac{\omega }{4\pi }{\varepsilon}_{2}(\omega )$$

**Fig. 16:**
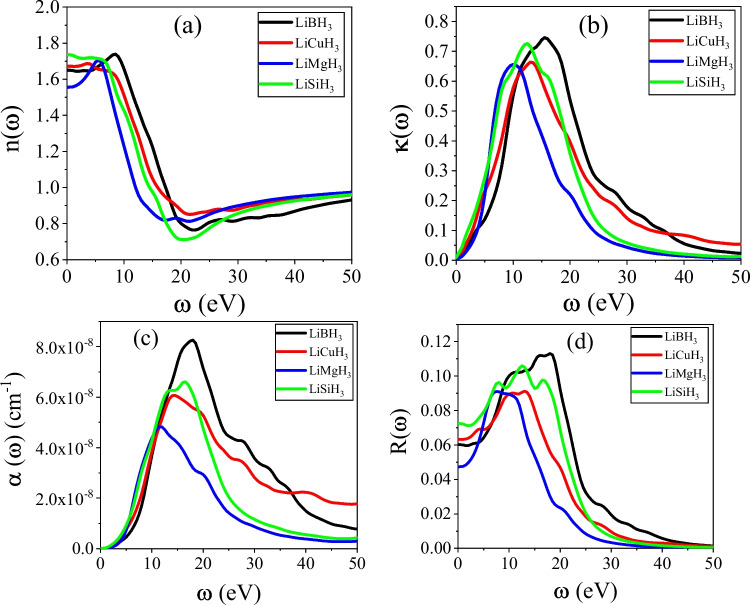
(**a**) refractive index, (**b**) extinction coefficient, (**c**) absorption coefficient, and (**d**) reflectivity of LiBH_3_, LiCuH_3_, LiMgH_3_, and LiSiH_3_ perovskite hydrides

The computed n(ω) of LiBH_3_, LiCuH_3_, LiMgH_3_, and LiSiH_3_ is shown in Fig. 16a. The calculated static refractive indices n(0) are 1.65 for LiBH_3_, 1.68 for LiCuH_3_, 1.56 for LiMgH_3_, and 1.74 for LiMgH_3_. The relatively large n(0) value of LiSiH_3_ is consistent with its high dielectric constant and strong lattice polarizability. Such enhanced polarizability is advantageous for hydrogen storage, as it allows the lattice to accommodate hydrogen atoms more effectively while maintaining structural stability during cycling.

The extinction coefficient $k\left(\omega \right)$, presented in Fig. 16b, describes the attenuation of electromagnetic waves within the material and directly correlates with ${\varepsilon}_{2}\left(\omega \right)$. Accordingly, the main peaks in $k\left(\omega \right)$ follow the same trend observed for the imaginary part of the dielectric function, confirming that absorption in these hydrides is dominated by interband electronic transitions.

Figure 16c illustrates the absorption coefficient $\alpha \left(\omega \right)$, which determines the penetration depth of incident radiation. All studied hydrides exhibit negligible absorption at zero frequency, consistent with their insulating or semiconducting nature. With increasing frequency, $\alpha \left(\omega \right)$ rises sharply, and strong absorption is observed primarily in the ultraviolet region (10–18 eV). The maximum absorption peaks occur at approximately 18.0 eV for LiBH_3_, 14.05 eV for LiCuH_3_, 11.67 eV for LiMgH_3_, and 16.47 eV for LiSiH_3_. High UV absorption indicates active electronic transitions that can support photon-induced or thermally assisted hydrogen release processes. In practical hydrogen storage systems, such behavior is beneficial because efficient absorption of radiation can induce localized heating or electronic excitation, thereby lowering the energy barrier for hydrogen desorption and improving storage–release kinetics [86].

The reflectivity spectra R(ω), shown in Fig. 16d, provide further insight into the surface optical response of the materials. The static reflectivity values R(0) are calculated to be 0.06, 0.063, 0.047, and 0.073 for LiBH_3_, LiCuH_3_, LiMgH_3_, and LiSiH_3_, respectively. The relatively higher reflectivity of LiSiH_3_ is indicative of strong interaction with incident photons and is consistent with its highly polarizable electronic structure. In hydrogen storage materials, such optical behaviour often correlates with efficient charge redistribution during hydrogen absorption and desorption, contributing to improved reversibility.

**Fig. 17:**
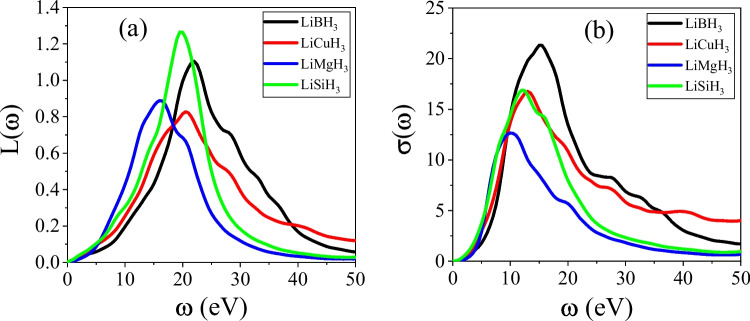
The computed (**a**) electron loss energy function and (**b**) conductivity for LiBH_3_, LiCuH_3_, LiMgH_3_, and LiSiH_3_ perovskite hydrides

The electron energy-loss function $L\left(\omega \right)$, depicted in Fig. 17a, characterizes the energy dissipated by fast electrons traversing the material and is closely related to plasmon excitations [18]. Prominent loss peaks are observed at about 22 eV for LiBH_3_, 20.5 eV for LiCuH_3_, 16.5 eV for LiMgH_3_, and 19.5 eV for LiSiH_3_. These plasmon energies reflect differences in valence electron density and bonding nature among the compounds. The comparatively higher loss peak of LiSiH_3_ suggests stronger collective electronic oscillations, which may influence hydrogen binding strength and stability within the lattice.

The optical conductivity $\sigma \left(\omega \right)$, shown in Fig. 17b, provides insight into photon-induced charge transport. LiMgH_3_ exhibits zero conductivity at zero photon energy, confirming its insulating character. With increasing photon energy, $\sigma \left(\omega \right)$, increases significantly for all hydrides, reaching maximum values of approximately 21.3 at 15.5 eV for LiBH_3_, 16.76 at 13.0 eV for LiCuH_3_, 12.66 at 10 eV for LiMgH_3_, and 16.89 at 12.0 eV for LiSiH_3_. The relatively high optical conductivity of LiBH_3_ indicates enhanced electronic mobility under photon excitation, which can facilitate hydrogen diffusion and improve adsorption–desorption kinetics [90]. However, the presence of imaginary phonon frequencies (as discussed in the vibrational analysis) may limit its practical applicability despite its favourable optical response.

### Hydrogen storage properties

Hydrogen is widely regarded as a key energy carrier for a sustainable energy economy due to its high gravimetric energy density and zero carbon emissions upon utilization [35]. Among solid-state storage materials, lightweight metal hydride perovskites have emerged as promising candidates owing to their high hydrogen content, tunable bonding characteristics, and structural flexibility [91]. The hydrogen storage performance of such materials is commonly assessed using two critical metrics: hydrogen storage capacity (gravimetric and volumetric) and hydrogen desorption temperature [92].

#### Hydrogen storage capacity

In hydride perovskites, hydrogen can be accommodated through chemisorption, involving strong metal–hydrogen bonding, and physisorption, associated with weaker interactions that enable reversibility [83]. For practical applications, both gravimetric capacity (hydrogen stored per unit mass) and volumetric capacity (hydrogen stored per unit volume) must be optimized.

Gravimetric capacity (${C}_{wt}$) is determined by the relation [93]:

36 $${C}_{wt}\%=\left(\frac{n{M}_{H}}{n{M}_{H}+{M}_{host}}\right)\times 100\%$$

In this equation, n is hydrogen to hydrides atomic ratio and ${M}_{H}$ and ${M}_{host}$ are molar mass of hydrogen and host compound, respectively.

**Fig. 18:**
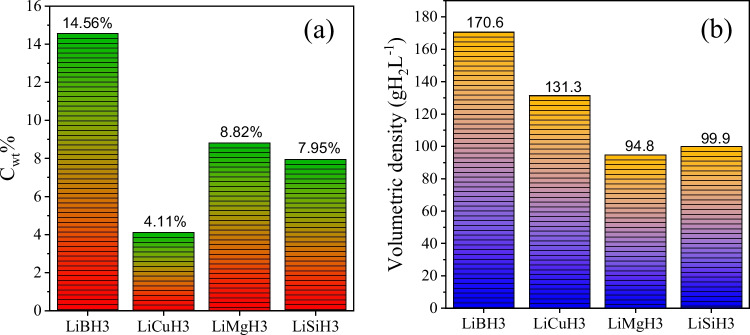
The calculated (**a**) gravimetric hydrogen storage capacities and (**b**) volumetric hydrogen density for LiBH_3_, LiCuH_3_, LiMgH_3_, and LiSiH_3_ perovskite hydrides

The calculated gravimetric hydrogen capacities of LiBH_3_, LiCuH_3_, LiMgH_3_, and LiSiH_3_ are 14.56, 4.11, 8.82, and 7.95 wt%, respectively (Fig. 18a). LiBH_3_ exhibits an exceptionally high hydrogen content, surpassing many DOE targets for solid-state hydrogen storage. However, its strong metal–hydrogen bonding also results in higher thermal stability, which can hinder hydrogen release. LiMgH_3_ and LiSiH_3_ offer a more balanced compromise between capacity and stability, making them attractive for reversible hydrogen storage. In contrast, the relatively low gravimetric capacity of LiCuH_3_ arises from the high atomic mass of Cu, although this is partially offset by its favorable thermodynamic stability.

In addition to gravimetric performance, volumetric hydrogen density is a crucial parameter for compact storage systems and is defined as [94, 95]:

37 $${V}_{\rho }=\frac{{N}_{H}{M}_{H}}{V\left(L\right){N}_{A}}$$

where ${N}_{H}$ is the number of hydrogen atoms per unit cell, $V\left(L\right)$ is the unit cell volume in liters, and ${N}_{A}$ is Avogadro’s number. The unit cell volumes obtained from DFT calculations are in Å^3^ and were converted to liters using: $1\mathrm{ \mathring{A} }^{3}=1\times {10}^{-27}L$. Thus, the volumetric hydrogen density is expressed in g L⁻^1^. All studied compounds crystallize in the cubic perovskite structure, for which the number of formula units per unit cell is Z = 1. Therefore, the total number of hydrogen atoms per unit cell is: ${N}_{H}=3\times Z=3$. This value was consistently used in all volumetric hydrogen density calculations.

As shown in Fig. 18b, LiBH_3_ exhibits the highest volumetric hydrogen density of 170.6 gH_2_ L⁻^1^, followed by LiCuH_3_, LiSiH_3_, and LiMgH_3_. The high volumetric density of LiBH_3_ reflects its compact crystal structure and high hydrogen concentration, whereas the lower values for LiMgH_3_ and LiSiH_3_ are associated with larger unit cell volumes. These results highlight the importance of simultaneously optimizing lattice density and atomic mass to achieve high overall storage efficiency.

#### Hydrogen desorption temperature

Beyond high storage capacity, practical hydrogen storage materials must release hydrogen at moderate temperatures with fast kinetics. The hydrogen desorption temperature ${T}_{des}$ defines the minimum temperature required for hydrogen release and is a key determinant of operational feasibility [18].

The hydrogen release reactions of LiXH_3_ (X = B, Cu, Mg, Si) perovskite hydrides can be expressed as:

38 $$LiX{H}_{3}\to Li+X+{~}^{3}\!\left/ \!{~}_{2}\right.{H}_{2}$$

where one formula unit of LiXH_3_ dissociates into one atom of Li, one atom of X, and 3/2 mol of H_2_ gas. The corresponding formation reaction is:

39 $$Li+X+{~}^{3}\!\left/ \!{~}_{2}\right.{H}_{2}\to LiX{H}_{3}$$

The formation enthalpy including zero-point energy (ZPE) correction is calculated as:

40 $$\Delta {H}_{f}={E}_{LiX{H}_{3}}-\left({E}_{Li}+{E}_{X}+{~}^{3}\!\left/ \!{~}_{2}\right.{E}_{{H}_{2}}\right)+ZPE$$

where ${E}_{LiX{H}_{3}}$, ${E}_{Li}$, ${E}_{X}$, ${E}_{{H}_{2}}$ are the DFT total energies at zero pressure and temperature, and ZPE ​ is the zero-point energy correction obtained from vibrational analysis.

Within a thermodynamic framework, the desorption temperature can be estimated from the Gibbs free energy relation [96]:

41 $$\Delta G=\Delta {H}_{f}-{T}_{des}.\Delta s$$

At equilibrium (ΔG=0), the desorption temperature is given by [97]:

42 $${T}_{des}=\frac{{\Delta H}_{f}}{\Delta S}$$

Here, ${\Delta H}_{f}$ is in J⋅mol^−1^ per mole of H_2_, and ΔS is the entropy change associated with hydrogen gas release, taken as 130.7 J⋅mol^−1^⋅K^−1^ per mole of H_2_ [32, 98]. Therefore, ${T}_{des}$ can be rewritten as:

43 $${T}_{des}=\frac{\left|{\Delta H}_{f}\right|}{130.7}$$

The calculated desorption temperatures for LiBH_3_, LiCuH_3_, LiMgH_3_, and LiSiH_3_ are 561.1, 457.7, 509.4, and 465.1 K, respectively. Among the studied compounds, LiCuH_3_ exhibits the lowest desorption temperature, closest to the desirable near-ambient range (233–333 K) for practical hydrogen delivery, indicating easier hydrogen release and favorable kinetics [99]. LiSiH_3_ shows a moderate desorption temperature, while LiBH_3_ and LiMgH_3_ require significantly higher temperatures due to stronger metal–hydrogen bonding.

The observed trend correlates well with the calculated formation energies. LiCuH_3_ possesses the least negative formation energy, implying weaker hydrogen binding and lower energetic barriers for absorption and desorption. Conversely, LiBH_3_ exhibits the most negative formation energy, consistent with its high gravimetric capacity but also its elevated desorption temperature. This trade-off between storage capacity and release temperature underscores the need for property optimization. Strategies such as chemical doping, strain engineering, or nanostructuring could effectively reduce desorption temperatures toward ambient conditions while preserving high hydrogen content.

Table 5 summarizes the hydrogen storage properties of the present Li-based hydride perovskites alongside previously reported systems. Compared with other DFT-studied hydride perovskites, LiBH_3_ stands out for its exceptional gravimetric and volumetric capacities, whereas LiCuH_3_ offers superior release characteristics. LiMgH_3_ and LiSiH_3_ occupy an intermediate regime, combining moderate storage capacity with acceptable desorption temperatures. These findings establish Li-based hydride perovskites as a versatile and tunable platform for next-generation solid-state hydrogen storage.

**Table 5:** DFT code and XC-functional used, desorption temperature (${T}_{des}$), gravimetric capacity (${C}_{wt}$), volumetric capacity (${V}_{\rho }$) of various metal hydrides studied by density functional theory for hydrogen storage

Hydrides	DFT code	XC-functional	${T}_{des}$(K)	${C}_{wt}$%	${V}_{\rho }$(g H_2_ L^−1^)	Ref
LiBH_3_	QE	PBE/PAW	561.1	14.56	170.6	This work
LiCuH_3_	QE	PBE/PAW	457.7	4.11	131.3	This work
LiMgH_3_	QE	PBE/PAW	509.4	8.82	94.8	This work
LiSiH_3_	QE	PBE/PAW	465.1	7.95	99.9	This work
LiPH_3_	WIEN2k	GGA-PBE	375.48	6.83	93.89	[6]
LiCaH_3_	VASP	PW91/PAW	453.76	5.99	63.77	[32]
LiSH_3_	CASTEP	GGA-PBE	−	7.19	75.59	[33]
LiAlH_3_	QE	PBE/MT	684.374	7.57	100.55	[34]
LiGaH_3_	QE	PBE/MT	785.516	3.66	95.44	[34]
LiScH_3_	CASTEP	PBE/USPP	−	5.7	−	[35]
LiTiH_3_	WIEN2k	GGA/PBEsol	−	5.22	−	[42]
LiRhH_3_	VASP	PBE/PAW	415.6	2.61	−	[43]
LiCaH_3_	QE	PBE/USPP	66.15	5.7	−	[44]
LiSrH_3_	CASTEP	PBE/PAW	−	3.098	−	[45]
LiZnH_3_	WIEN2k	GGA-PBE	435.5	4.02	131.0	[47]
LiVH_3_	WIEN2k	GGA-PBE	1904	4.97	−	[48]
LiNiH_3_	VASP	PBE/PAW	446.3	4.40	−	[93]
LiMnH_3_	CASTEP	PBE/NC	585	4.65	180.80	[100]

#### Elemental origin of hydrogen storage performance

To provide a systematic understanding of the hydrogen storage behavior, the differences among LiBH_3_​, LiCuH_3_​, LiMgH_3_​, and LiSiH_3_ can be directly related to the intrinsic characteristics of the B, Cu, Mg, and Si atoms. First, the atomic mass strongly influences the gravimetric hydrogen capacity. Boron, being the lightest element among the four constituents, leads to the highest gravimetric hydrogen storage capacity (14.56 wt%), whereas the heavy Cu atom significantly reduces the hydrogen weight fraction to 4.11 wt%. Mg and Si possess intermediate atomic masses, resulting in moderate capacities of 8.82 and 7.95 wt%, respectively. Second, differences in atomic size and packing efficiency affect the volumetric hydrogen density. The relatively small atomic radius of B promotes a compact crystal lattice and consequently the highest volumetric hydrogen density, while the larger Mg and Si atoms expand the unit cell and reduce volumetric storage performance.

Third, the electronic configuration and electronegativity of the constituent elements govern the metal–hydrogen bond strength and, therefore, the hydrogen release temperature. Boron forms highly directional covalent B–H bonds due to its high electronegativity, resulting in strong hydrogen binding and the highest desorption temperature. In contrast, Cu, with its filled 3d^10^ electronic configuration and weaker affinity toward hydrogen, forms comparatively weaker Cu–H interactions, facilitating hydrogen release at lower temperatures. Mg exhibits predominantly ionic Mg–H interactions that provide a balance between storage capacity and thermal stability, whereas Si forms mixed ionic–covalent Si–H bonds, yielding intermediate desorption behavior. Therefore, the hydrogen storage performance is governed by a fundamental trade-off between low atomic mass, crystal packing density, and hydrogen binding strength. While B optimizes hydrogen capacity, Cu improves hydrogen release characteristics, and Mg and Si provide a compromise between storage capacity and operating temperature. This analysis demonstrates that careful elemental substitution at the X-site in LiXH_3_ offers an effective route for tailoring the hydrogen storage properties of Li-based perovskite hydrides.

## Conclusions

In this work, a comprehensive first-principles (DFT) investigation was carried out to evaluate the structural, dynamical, mechanical, electronic, optical, thermodynamic, and hydrogen storage properties of LiBH_3_, LiCuH_3_, LiMgH_3_, and LiSiH_3_ perovskite hydrides. Structural optimization yielded lattice parameters between 3.087 and 3.756 Å, reflecting the influence of cation size on crystal geometry. All compounds exhibit negative formation energies, confirming their thermodynamic stability and potential synthesizability. Dynamical stability analysis shows that LiBH_3_, LiCuH_3_, and LiMgH_3_ are free from imaginary phonon frequencies, whereas LiSiH_3_ is dynamically unstable. Mechanical analysis confirms that all compounds satisfy the Born stability criteria, with LiCuH_3_ and LiMgH_3_ exhibiting higher elastic stiffness and improved resistance to deformation. Electronic structure calculations reveal metallic behavior for LiBH_3_, LiCuH_3_, and LiSiH_3_, while LiMgH_3_ is semiconducting with a bandgap of 2.59 eV. Optical properties indicate strong dielectric response and absorption in LiCuH_3_ and LiSiH_3_, suggesting their suitability for energy-related applications. Hydrogen storage analysis shows gravimetric capacities of 14.56 wt% for LiBH_3_, 4.11 wt% for LiCuH_3_, 8.82 wt% for LiMgH_3_, and 7.95 wt% for LiSiH_3_, with LiBH_3_ exhibiting the highest storage capacity, making it particularly attractive for high-capacity hydrogen storage applications. Additionally, LiCuH_3_ presents the lowest hydrogen desorption temperature (457.7 K), indicating favorable release kinetics.

## Data Availability

The data that support the ﬁndings of this study are available from the corresponding author upon reasonable request.
